# Supplementary material for: Older adults’ experiences and perceived impacts of the Aging, Community and Health Research Unit-Community Partnership Program (ACHRU-CPP) for diabetes self-management in Canada: a qualitative descriptive study
Source: BMJ Open. 2023 Apr 5;13(4):e068694. doi: 10.1136/bmjopen-2022-068694 (PMC10083734; doi:10.1136/bmjopen-2022-068694)
Supplement: Author's manuscript [file bmjopen-2022-068694.draft_revisions.pdf]

BMJ Open is committed to open peer review. As part of this commitment we make the peer review history of every article we publish publicly available.

When an article is published we post the peer reviewers' comments and the authors' responses online. We also post the versions of the paper that were used during peer review. These are the versions that the peer review comments apply to.

The versions of the paper that follow are the versions that were submitted during the peer review process. They are not the versions of record or the final published versions. They should not be cited or distributed as the published version of this manuscript.

BMJ Open is an open access journal and the full, final, typeset and author-corrected version of record of the manuscript is available on our site with no access controls, subscription charges or pay-per-view fees (http://bmjopen.bmj.com).

If you have any questions on BMJ Open's open peer review process please email info.bmjopen@bmj.com

BMJ Open

Older Adults' Experiences and Perceived Impacts of the Aging, Community and Health Research Unit – Community Partnership Program (ACHRU-CPP) for Diabetes Self-Management

Journal:	*BMJ Open*
Manuscript ID	bmjopen-2022-068694
Article Type:	Original research
Date Submitted by the Author:	27-Sep-2022

Complete List of Authors:	Yous, Marie-Lee; McMaster University Faculty of Health Sciences, School of Nursing<br>Ganann, Rebecca; McMaster University Faculty of Health Sciences, School of Nursing, Aging, Community and Health Research Unit<br>Ploeg, Jenny ; McMaster University Faculty of Health Sciences, School of Nursing, Aging, Community and Health Research Unit<br>Markle-Reid, Maureen; McMaster University Faculty of Health Sciences, School of Nursing, Aging, Community and Health Research Unit<br>Northwood, Melissa ; McMaster University Faculty of Health Sciences, School of Nursing, Aging, Community and Health Research Unit<br>Fisher, Kathryn ; McMaster University School of Nursing, School of Nursing, Aging, Community and Health Research Unit<br>Valaitis, Ruta; McMaster University Faculty of Health Sciences, School of Nursing, Aging, Community and Health Research Unit<br>Chambers, Tracey; McMaster University Faculty of Health Sciences, School of Nursing, Aging, Community and Health Research Unit<br>Montelpare, William; University of Prince Edward Island, Department of Applied Human Sciences, Faculty of Science<br>Légaré, France; VITAM Centre de recherche en santé durable; Laval University, Department of Family and Emergency Medicine, Faculty of Medicine<br>Beleno, Ron; McMaster University Faculty of Health Sciences, School of Nursing, Aging, Community and Health Research Unit<br>Gaudet, Gary; University of Prince Edward Island<br>Giacometti, Luisa; McMaster University Faculty of Health Sciences, School of Nursing, Aging, Community and Health Research Unit<br>Levely, Deborah; McMaster University Faculty of Health Sciences, School of Nursing, Aging, Community and Health Research Unit<br>Lindsay, Craig; McMaster University Faculty of Health Sciences, School of Nursing, Aging, Community and Health Research Unit<br>Morrison, Allan; University of Prince Edward Island

Keywords:	QUALITATIVE RESEARCH, General diabetes < DIABETES & ENDOCRINOLOGY, PRIMARY CARE

I, the Submitting Author has the right to grant and does grant on behalf of all authors of the Work (as defined in the below author licence), an exclusive licence and/or a non-exclusive licence for contributions from authors who are: i) UK Crown employees; ii) where BMJ has agreed a CC-BY licence shall apply, and/or iii) in accordance with the terms applicable for US Federal Government officers or employees acting as part of their official duties; on a worldwide, perpetual, irrevocable, royalty-free basis to BMJ Publishing Group Ltd ("BMJ") its licensees and where the relevant Journal is co-owned by BMJ to the co-owners of the Journal, to publish the Work in this journal and any other BMJ products and to exploit all rights, as set out in our licence.

The Submitting Author accepts and understands that any supply made under these terms is made by BMJ to the Submitting Author unless you are acting as an employee on behalf of your employer or a postgraduate student of an affiliated institution which is paying any applicable article publishing charge ("APC") for Open Access articles. Where the Submitting Author wishes to make the Work available on an Open Access basis (and intends to pay the relevant APC), the terms of reuse of such Open Access shall be governed by a Creative Commons licence – details of these licences and which Creative Commons licence will apply to this Work are set out in our licence referred to above.

Other than as permitted in any relevant BMJ Author's Self Archiving Policies, I confirm this Work has not been accepted for publication elsewhere, is not being considered for publication elsewhere and does not duplicate material already published. I confirm all authors consent to publication of this Work and authorise the granting of this licence.

For peer review only

BMJ Open

Original Research

Title: Older Adults' Experiences and Perceived Impacts of the Aging, Community and Health Research Unit – Community Partnership Program (ACHRU-CPP) for Diabetes Self-Management

Corresponding Author: Marie-Lee Yous, School of Nursing, Aging, Community and Health Research Unit, Faculty of Health Sciences, McMaster University, 1280 Main Street West, Room HSC 3N25, Hamilton, Ontario L8S 4K1, Canada, Email: yousm@mcmaster.ca; ORCID: 0000-0002-4271-0401

Marie-Lee Yous, School of Nursing, Aging, Community and Health Research Unit, Faculty of Health Sciences, McMaster University, Hamilton, Ontario, Canada, email: yousm@mcmaster.ca ORCID: 0000-0002-4271-0401

Rebecca Ganann, School of Nursing, Aging, Community and Health Research Unit, Faculty of Health Sciences, McMaster University, email: gananrl@mcmaster.ca ORCID: 0000-0002-7566-8932

Jenny Ploeg, School of Nursing, Aging, Community and Health Research Unit, Faculty of Health Sciences, McMaster University, Hamilton, Ontario, Canada, email: ploegj@mcmaster.ca ORCID: 0000-0001-8168-8449

Maureen Markle-Reid, School of Nursing, Aging, Community and Health Research Unit, Faculty of Health Sciences, McMaster University, Hamilton, Ontario, Canada, email: mreid@mcmaster.ca ORCID: 0000-0002-4019-7077

Melissa Northwood, School of Nursing, Aging, Community and Health Research Unit, Faculty of Health Sciences, McMaster University, Hamilton, Ontario, Canada, email: northwm@mcmaster.ca ORCID: 0000-0001-5043-8068

Kathryn Fisher, School of Nursing, Aging, Community and Health Research Unit, Faculty of Health Sciences, McMaster University, Hamilton, Ontario, Canada fisheka@mcmaster.ca ORCID: 0000-0001-8342-1238

Ruta Valaitis, School of Nursing, Aging, Community and Health Research Unit, Faculty of Health Sciences, McMaster University, Hamilton, Ontario, Canada, email: valaitis@mcmaster.ca ORCID: 0000-0002-3117-0542

Tracey Chambers, School of Nursing, Aging, Community and Health Research Unit, Faculty of Health Sciences, McMaster University, Hamilton, Ontario, Canada, email: chambt@mcmaster.ca ORCID: 0000-0002-9325-6894

William Montelpare, Margaret and Wallace McCain Chair in Human Development and Health, Department of Applied Human Sciences, Faculty of Science, University of Prince Edward

For peer review only - http://bmjopen.bmj.com/site/about/guidelines.xhtml

Island, Charlottetown, Prince Edward Island, Canada, email: wmontelpare@upei.ca ORCID: 0000-0002-4167-4613

France Légaré, VITAM-Centre de recherche en santé durable, Université Laval, Québec City, Québec, Canada, email: France.legare@fmed.ulaval.ca ORCID: 0000-0002-2296-6696

Ron Beleno, Patient/Public Research Partner, Aging, Community and Health Research Unit, Faculty of Health Sciences, McMaster University, Hamilton, Ontario, Canada email: ron@rb33.com

Gary Gaudet, Patient/Public Research Partner, University of Prince Edward Island, Charlottetown, Prince Edward Island, Canada, email: l.ggaudet@bellaliant.net

Luisa Giacometti, Patient/Public Research Partner, School of Nursing, Aging, Community and Health Research Unit, Faculty of Health Sciences, McMaster University, Hamilton, Ontario, Canada, email: luisag@bell.net

Deborah Levely, Patient/Public Research Partner, School of Nursing, Aging, Community and Health Research Unit, Faculty of Health Sciences, McMaster University, Hamilton, Ontario, Canada, email: deborah.levely@rogers.com

Craig Lindsay, Patient/Public Research Partner, School of Nursing, Aging, Community and Health Research Unit, Faculty of Health Sciences, McMaster University, Hamilton, Ontario, Canada, email: lindsay.cr@gmail.com

Allan Morrison, Patient/Public Research Partner, University of Prince Edward Island, Charlottetown, Prince Edward Island, Canada, email: allan.morrison62.am@gmail.com

On behalf of the ACHRU-CPP Research Team

Word count excluding title page, abstract, references, figures and tables **(max 4,000**): 4,000

For peer review only

For peer review only - http://bmjopen.bmj.com/site/about/guidelines.xhtml

BMJ Open

Abstract (max 300 words): 299 words

Objectives: The aim of this study was to assess the experiences and perceived impacts of the Aging, Community and Health Research Unit – Community Partnership Program (ACHRU-CPP) from the perspectives of older adults with diabetes and other chronic conditions. The ACHRU-CPP is a complex 6-month self-management evidence-based intervention for community-living older adults aged 65 years or older with Type 1 or Type 2 diabetes and at least one other chronic condition. Its components include home and phone visits, care coordination, system navigation support, caregiver support, and group wellness sessions delivered by a nurse, dietitian or nutritionist, and community program coordinator to meet older adults' health and social needs.

Design: A qualitative descriptive design embedded within a randomized controlled trial was used.

Setting: Six trial sites offering primary care services from three Canadian provinces (i.e., Ontario, Quebec, and Prince Edward Island) were included.

Participants: The sample consisted of 45 community-living older adults aged 65 years or older with diabetes and at least one other chronic condition.

Methods: Participants completed semi-structured post-intervention interviews that were completed by phone in English or French. The analytical process followed Braun and Clarke's experiential thematic analysis framework. Patient partners informed study design and interpretation.

Results: Older adults reported positive experiences with the ACHRU-CPP that supported diabetes self-management such as, improved knowledge in managing diabetes and other chronic conditions, enhanced physical activity and function, improved eating habits, and opportunities

For peer review only - http://bmjopen.bmj.com/site/about/guidelines.xhtml

for socialization. They reported being connected to community resources by the intervention team to address social determinants of health and support self-management practices.

Conclusions: Older adults perceived that a 6-month person-centred intervention collaboratively delivered by a team of health and social care providers helped support chronic disease self-management. There is a need for providers to help older adults connect with available health and social services in the community.

Strengths and limitations of this study

- This study included a rigorous qualitative design with a large sample size.
- There was the inclusion of diverse participants with regards to sex, marital status, and annual income from multiple sites across Canada.
- A limitation of the study is the lack of cultural diversity with regards to ethnicity and under-representation of older adults from marginalized communities.

Keywords: diabetes self-management, older adults, community, primary care, qualitative

For peer review only - http://bmjopen.bmj.com/site/about/guidelines.xhtml

Introduction

As of 2022, approximately 422 million people have been diagnosed with diabetes mellitus worldwide [1]. Older adults are more likely to have Type 2 diabetes than younger adults [2] and are at risk for hypoglycemia which can adversely affect cognition, vision, hearing, mobility, and mental health [3] as well as self-care activities including exercise and diet. More than 40% of older adults with diabetes have three or more chronic conditions [4], including hyperlipidemia, hypertension, asthma, chronic obstructive pulmonary disease, chronic kidney disease, arthritis, and heart failure [5]. Following management plans for one condition may be challenging due to symptoms or conflicting guidelines from another condition. Higher burden associated with the presence of multiple chronic conditions (MCC) has been linked to higher risk for mortality, decreased physical and mental functioning, and increased health services use [2, 6, 7]. Community-dwelling older adults with MCC are highly reliant on family/friend caregivers for support [8], which can lead to poor mental and physical health, and financial losses among caregivers [9]. Caregivers' unmet needs [10] can lead to their increased use of hospital and emergency services.

Complex health interventions are defined as having multiple interacting components [11]. For older adults with diabetes, complex health interventions such as peer support programs, have demonstrated positive effects in managing their complex needs, sustaining lifestyle changes, and achieving health benefits [12-17]. Complex interventions that target self-care and incorporate opportunities for peer-to-peer discussions among community-dwelling older adults can improve their mental and physical health and reduce falls [18, 19]. Nurse-led self-management programs for diabetes and other chronic conditions can lead to improvements in self-rated health, glycated hemoglobin (HbA1c) values, blood pressure, weight, and self-management behaviours [19, 20].

For peer review only - http://bmjopen.bmj.com/site/about/guidelines.xhtml

Providing diabetes self-management programs through partnerships between primary care and community organizations (e.g., senior centres, YMCA) supports program uptake, implementation, and sustainability [12], which can lead to improved health literacy (i.e., being able to locate, read and understand health information for informed decision-making). In a systematic review of randomized controlled trials (RCTs), health literacy was instrumental in enhancing diabetes knowledge, self-efficacy, and physical activity [21].

Receiving care from multiple providers from different health and community providers can lead to fragmented care for older adults, as referrals across organizations are often not well integrated [22]. Seamless care coordination and system navigation for older adults with MCC remain high priorities for this population. There is a need for innovative programs for older adults that focus on the Quintuple Aims of high-quality care: (a) enhancing the patient experience of care; (b) creating healthy populations; (c) reducing healthcare costs; (d) improving the care delivery experience; and (e) health equity [23]. Gaps in previous intervention designs include lack of emphasis on patient experience of care and considerations for health equity [23].

The Aging, Community and Health Research Unit – Community Partnership Program (ACHRU-CPP) is a 6-month self-management intervention for community-living older adults (≥ 65 years old), diagnosed with Type 1 or Type 2 diabetes and at least one other chronic condition, and their family/friend caregivers (≥ 18 years old). The intervention was evaluated in a feasibility study in Ontario, Canada [24], followed by a clinical trial in selected primary care and community settings in two Canadian provinces (Ontario and Alberta) [25-27]. A multi-jurisdictional pragmatic RCT is currently in progress to evaluate the implementation and effectiveness of the ACHRU-CPP in three Canadian provinces. To better understand how to

For peer review only

For peer review only - http://bmjopen.bmj.com/site/about/guidelines.xhtml

address the complex needs of older adults, we sought to assess the experiences and perceived impacts of the ACHRU-CPP from the perspectives of older adults with diabetes and MCC.

Methods

Design

This qualitative study is embedded within the multi-site implementation-effectiveness type II hybrid RCT, as outlined in the protocol paper [28]. This study used a qualitative descriptive design, as described by Sandelowski [29, 30], to provide a fulsome summary while remaining close to the words of participants when describing their experiences with the ACHRU-CPP and its perceived impacts.

ACHRU-CPP Program

The ACHRU-CPP is delivered by an interprofessional team of primary care providers, which includes a Registered Nurse (RN) and Registered Dietitian (RD) or Nutritionist from a primary care setting, and a Program Coordinator (e.g., Registered Kinesiologist) from a local community partner organization (hereafter referred to as the intervention team). Table 1 summarizes the core components of the ACHRU-CPP. Due to the COVID-19 pandemic some participants received virtual visits by phone or videoconferencing. A comparison of results from the virtual and in-person approaches will be published in a future paper.

Table 1. The ACHRU-CPP five core components

Intervention Components	Goals
1. Home/virtual visits (up to 3 home visits) and unlimited follow-up phone calls by a RN and/or RD or Nutritionist	To assess older adults' and caregivers' needs and goals using standardized tools to support a coordinated care plan
2. Monthly group wellness sessions (up to 6 sessions) at a local community centre led by the RN, RD or Nutritionist and Community Program Coordinator	To provide older adults and caregivers with gentle progressive physical activity, self-management education for diabetes and other chronic conditions, and healthy lunches and snacks

For peer review only - http://bmjopen.bmj.com/site/about/guidelines.xhtml

Intervention Components	Goals
3. Monthly team case conferences which include a RN, RD or Nutritionist, and Community Program Coordinator	To discuss the health and social care needs of older adults and caregivers, develop and revise the coordinated care plan, and plan topics for group wellness sessions
4. Collaboration with the primary care interprofessional team and other specialists (e.g., family physicians, nurse practitioners, kinesiologists, social workers, home care and social service providers, pharmacists, endocrinologists)	To support primary care and community providers in working collaboratively to develop care plans for older adults, and connect older adults and caregivers to specialists and community resources
5. Nurse-led care coordination/system navigation	To facilitate linkages to other primary healthcare providers, specialists and community care services for older adults and caregivers

Note. RN = Registered Nurse; RD = Registered Dietitian

Patient and Public Involvement

The need for the program was originally identified by community-dwelling older adults with diabetes and other chronic conditions and their caregivers and was subsequently co-designed by older adults in collaboration with primary and community care providers and researchers [24]. In the current RCT, patient partners from the pan-Canadian Steering Committee were involved in reviewing research questions and advising the research team on the selection of outcome measures [28]. Patient and public research partners also participated in local Community Advisory Boards in each site to inform further adaptations to the intervention, and support local implementation. Patient and public research partners from the local community advisory boards (RB, GG, LG, CL, DL, AM) and the Steering Committee (LM, FT) also provided input into development of this manuscript by reviewing and interpreting the results and helping to shape the key messages.

Setting

The study was conducted in two sites in Ontario, Quebec, and Prince Edward Island, Canada. Each of the six sites was selected to ensure variability in geographic setting (urban and

For peer review only

For peer review only - http://bmjopen.bmj.com/site/about/guidelines.xhtml

BMJ Open

rural); socio-demographic and cultural backgrounds; language spoken (English or French); demonstrated support for the ACHRU-CPP; and the presence of staff to support intervention implementation. An RN and RD or Nutritionist from a primary care setting or diabetes education program worked in partnership with a program coordinator from a local community partner site (e.g., YMCA) to implement the program.

Sample and Recruitment

Older adults were screened for eligibility to participate in the RCT by a trained staff member of the primary care site, as described in the study protocol [28]. Eligible patients met the following inclusion criteria: (a) aged 65 years or older; (b) diagnosis of Type 1 or Type 2 diabetes with at least one other chronic condition; (c) receiving primary care services from one of the participating primary care settings; (d) living within the area served by the primary care setting and community site; (f) able to provide informed consent or has a substitute decision-maker able to provide informed consent on the patient's behalf; and (g) competent in English or French, or has an interpreter competent in English or French.

Following the completion of baseline interviews, patients were randomized to receive the intervention (i.e., ACHRU-CPP) in addition to usual care or usual care alone. A total of 8-10 older adults per site who completed the 6-month intervention were invited to participate in follow-up telephone interviews. Trained research assistants (MY, RC), with no prior relationship with participants, used a telephone script to call selected older adults within two weeks of completing the ACHRU-CPP, to invite them to participate in a telephone interview. Maximum variation purposive sampling [31] was used to select a diverse sample of participants across all 6 sites based on their sex, annual income, ethnicity, and level of participation in all components of the study.

For peer review only - http://bmjopen.bmj.com/site/about/guidelines.xhtml

225 **Data Collection**

226 Semi-structured post-intervention telephone interviews were conducted between April 2020 and August 2021. Trained research assistants conducted audio-recorded interviews, ranging from 20 to 60 minutes in length, in English or French. Interviews were transcribed verbatim by experienced transcriptionists. Interviews conducted in English were transcribed and cleaned by trained research staff, while interviews conducted in French were transcribed and translated into English by professional transcriptionists and later validated by a bilingual member of the research team. Transcripts were not returned to participants for their review. The interview guide was created based on: (a) a review of the literature of health and social needs of older adults and caregivers, patient-provider communication, and system navigation and (b) feedback from patient partners and the research team with expertise in aging, community-based supports for older adults and caregivers, and qualitative research. Table 2 provides sample interview questions.

238 **Table 2.** Sample interview questions for older adults

**Questions for Older Adults**
1. What did you need the most in the past six months (e.g., physical, emotional, mental or psychological support, transportation, financial assistance, housekeeping, personal care support)?
2. When [name of nurse and dietitian] visited you what types of things did they do during those visits?
3. For other people who are living with diabetes and other chronic conditions, would you recommend that a nurse or dietitian, such as [name of nurse and dietitian], visit the person at home, make phone calls or both?
4. What types of things did you do at the monthly wellness sessions or during the individual calls with [name of the community program coordinator]?
5. How, if at all, did [name of nurse and dietitian] involve you in decisions about your care?

For peer review only - http://bmjopen.bmj.com/site/about/guidelines.xhtml

6. How, if at all, did [name of nurse and dietitian] help you to connect with other community health or social services to help you?
7. To what extent did the nurse and dietitian help to address your needs or the issues that were most important to you?
8. How happy are you with the overall care that you received from [name of nurse and dietitian]?
9. Was the information given by [name of nurse and dietitian] and other health professionals about care consistent (across individuals)?
10. Is there anything else about your experiences with [name of nurse and dietitian] that you would like to add that we haven't already discussed?

Data Analysis

Themes were generated using the Braun and Clarke's experiential thematic analysis framework [32] and organized under relevant constructs of the Consolidated Framework for Implementation Research framework. [33]. Thematic analysis was selected to ensure that the development of themes was informed by the experiences and perceived intervention benefits of older adults. The six phases of thematic analysis include: (a) becoming familiar with the data; (b) coding; (c) developing themes; (d) reviewing themes; (e) constructing a definition for themes and labelling them; and (f) creating a report [32]. A female research assistant with doctoral level training in qualitative research (MY) used the data management software NVivo version 12 [34] to perform coding. MY is fluent in both languages and coded in English. Results were shared with the team in English only. Following the creation of codes, these were further examined for patterns to generate themes. Themes were shared with the research team, including patient partners, to ensure they were reflective of the data.

Rigour and Trustworthiness

Consensus was reached by all authors prior to the inclusion of themes in the final report.

For peer review only - http://bmjopen.bmj.com/site/about/guidelines.xhtml

Lincoln and Guba's validation criteria [35] were applied in this study to enhance the study's rigour. To support the credibility of findings, investigator triangulation was used in data analysis through team meetings with 5-7 members to review the coding structure and evidence of themes. These members included patient and public research partners and researchers of various disciplines with expertise in qualitative research, gerontology, and community-based interventions. Conflicts were resolved through team consensus. To facilitate transferability of findings, the study sample and setting were described in detail. To support dependability and confirmability of findings, the research team kept an audit trail of study processes.

Results

A total of 295 older adults were enrolled in the RCT and randomly allocated to receive the ACHRU-CPP or usual care. At the time of data collection, 53 older adults who had completed the 6-month intervention were approached to participate in the qualitative interviews and 45 accepted (84.91%).

Demographic Characteristics

The mean age of the 45 older adults who participated in interviews was 71.1 years. Most were female (55.6%), retired from paid work (80%), had Type 2 diabetes (93.3%), and reported 4-6 chronic conditions (44.4%). Hypertension, hyperlipidemia, and osteoarthritis and other arthritis (e.g., rheumatoid arthritis) were the most reported chronic conditions. Table 3 summarizes demographic characteristics of participants.

Table 3. Demographic characteristics of interview participants

**Older Adults (n=45)**	
**Category**	**n (%)**
Age (mean [Standard Deviation]): 71.7 [6.5]	
65-70	26 (57.8)
71-75	7 (15.6)
76+	12 (26.7)

For peer review only - http://bmjopen.bmj.com/site/about/guidelines.xhtml

Older Adults (n=45)	
**Category**	**n (%)**
Sex	
Female	25 (55.6)
Male	20 (44.4)
Marital Status	
Married or living with a partner	21 (46.7)
Divorced, never married, separated, or widowed	23 (51.1)
Refused	1 (2.2)
Highest Level of Education	
Completed a graduate or professional degree	6 (13.3)
Completed a bachelor's degree	10 (22.2)
Had some university education or completed a community college, technical college, or postsecondary program	12 (26.7)
Completed secondary school	10 (22.2)
Did not complete secondary school	7 (15.6)
Current Employment Status	
Retired from paid work	36 (80.0)
Employed full-time	4 (8.9)
Employed part-time	2 (4.4)
Unemployed and looking for work	1 (2.2)
Refused	2 (4.4)
Annual Household Income	
$150,000 or more	2 (4.4)
$100,000 or more, but less than $150,000	2 (4.4)
$50,000 or more, but less than $100,000	12 (26.7)
$20,000 or more, but less than $50,000	16 (35.6)
Less than $20,000	11 (24.4)
Refused	2 (4.4)
Born in Canada	
Yes	31 (68.9)
Ethnic/Racial Group	
White/Caucasian	32 (71.1)
Black	3 (6.7)
Caribbean/Guyanese	3 (6.7)
Filipino	2 (4.4)
First Nations	1 (2.2)
South Asian	1 (2.2)
Southeast Asian	1 (2.2)
Chinese	1 (2.2)
Japanese	1 (2.2)
Language(s) Spoken	
English	37 (82.2)
French	15 (33.3)

For peer review only - http://bmjopen.bmj.com/site/about/guidelines.xhtml

Older Adults (n=45)	
**Category**	**n (%)**
Living with Others (e.g., spouse, children, other relative, friend, group setting)	
Yes	27 (60.0)
Type of Diabetes	
Type 1 diabetes	1 (2.2)
Type 2 diabetes	42 (93.3)
Unknown	2 (4.4)
Number of Chronic Conditions (mean [Standard Deviation]): 5.6 [2.9]	
1-3	11 (24.4)
4-6	20 (44.4)
7-9	8 (17.8)
10 +	6 (13.3)
Commonly Reported Chronic Conditions	
Hypertension	34 (75.6)
Hyperlipidemia	27 (60.0)
Osteoarthritis and other arthritis	18 (40.0)
Cardiovascular disease	16 (35.6)
At Least 1 Emergency Room Visit in the Last 6 Months	
6 months prior to ACHRU-CPP	8 (17.8)
6-month follow-up	7 (15.6)

Themes

Themes were grouped into two categories, experiences, and perceived impacts of the ACHRU-CPP. Table 4 provides an overview of themes. The words in italics that label the theme are taken verbatim from transcripts. Similarly, participant quotes in the narrative that follows are noted in italics and identified by OA for older adult, # for site number, and ### for participant number.

Table 4. Themes of older adult experiences and perceived impacts with the ACHRU-CPP

**Experiences with the ACHRU-CPP**
• In-depth dialogue with "*professional friends*"<br>• Socialized with *"people with the same type of health problems"*<br>• Person-centred care by *"more than one knowledgeable person"*

For peer review only - http://bmjopen.bmj.com/site/about/guidelines.xhtml

• Ongoing contact with providers so *"you are not alone"*<br>• Need to address ethnic/cultural differences through a *"personal session"*
**Perceived Impacts of the ACHRU-CPP**
• Improved diabetes self-management behaviours: *"make more proactive steps"*<br>• Added connection to health and social support services *"that could help me"*

Experiences with the ACHRU-CPP

Overall, older adults reported positive experiences with the ACHRU-CPP. They experienced: (a) in-depth dialogue with "*professional friends"*; (b) socialized with *"people with the same type of health problems"*; (c) person-centred care by *"more than one knowledgeable person"; (d)* ongoing contact with providers so *"you are not alone";* and identified the (e) need to address ethnic/cultural differences: *"eating has to do with seasons"*.

In-depth dialogue with *"professional friends"*. In-person home and virtual visits were perceived by older adults as more relaxed compared to clinic visits and provided opportunities for in-depth dialogue about health and social issues with providers. "*The home visits are more relaxed, if you were at a clinic, you got a time slot you got to meet whatever is transacted in that timeframe"* (OA_1_152). Home visits helped to build trust between older adults and the providers, which facilitated the exploration of concerns and needs beyond diabetes such as safe housing and transportation issues. "*I felt I could trust her [nutritionist]*" (OA_5_037). Providers were approachable and understanding of older adults' situations and were considered as friends and confidants. *"They [providers] were professional friends"* (OA_2_242). Their approach was especially important when discussing sensitive topics such as mental health concerns. "*I had my*

For peer review only - http://bmjopen.bmj.com/site/about/guidelines.xhtml

BMJ Open

sick niece calling me and that was stressing me, so she [nurse] said to "Let go. When we are stressed, that's not always good". So how to manage my stress" (OA_6_023).

Socialized with *"people with the same type of health problems".* Older adults and caregivers perceived that group wellness sessions helped them meet others who understood what it is like to live with diabetes and other chronic conditions. *"The fact of socializing with other people with the same types of health problems as we do"* (OA_5_037). The sessions provided opportunities for group exercises which provided peer motivation. The group sessions were particularly helpful for older adults who were socially isolated, and some older adults became friends because of the sessions.

Person-centred care by *"more than one knowledgeable person".* Older adults appreciated that they received person-centred care from a team of providers through the ACHRU-CPP to discuss diabetes, their other chronic conditions, and social concerns. Older adults valued providers working collaboratively to meet their needs. *"It was good that they worked in a team. More than one knowledgeable person. That was important"* (OA_2_242). They felt that providers were listening to their concerns and that, prior to meeting with the intervention team, it was difficult to find the right person to talk to about diabetes.

> *I enjoyed having them come to visit. I don't talk to a lot of people about my diabetes because I don't feel it's that complicated, but nobody really wants to listen about your health issues.* (OA_3_032)

Providers supported the management of other conditions in addition to diabetes. *"I was having troubles with my bowels, but we got that regulated and it's good"* (OA_4_075).

Ongoing contact with providers so *"you are not alone".* Providers made older adults feel that someone was concerned about their well-being. "*It's not as if we are just left alone with our problems. What you are doing is very good; continue"* (OA_5_128). Follow-up phone calls

For peer review only

For peer review only - http://bmjopen.bmj.com/site/about/guidelines.xhtml

were well received by older adults, especially by those living alone or with little support, and ensured that they “*haven't fallen through the cracks*” (OA_3_058). The ongoing follow-up with the team reinforced familiar information that older adults had forgotten to put into practice over time.

Need to address ethnic/cultural differences through a *“personal session”*. Some ethnic groups may have language barriers and be *“very shy and they don't approach people unless someone else pushes them to go [join programs]”* (OA_1_061). Some older adults perceived that individual wellness sessions with providers may be helpful for those with language barriers. *“Some of them had a bit of a language problem. I think a personal session would be much more helpful”* (OA_2_086). There is a need to allow dedicated time for older adults to share their cultural practices during interactions with peers and the intervention team.

> *One of the things I learned about myself from my community [Indigenous community] and my family is that eating has to do with seasons...Your year-round diet has to do with what's available to you...I mentioned that one time in the group [Group Wellness Sessions]and they thought that had nothing to do with what the topics were.* (OA_2_013)

Perceived Impacts

Older adults perceived that the ACHRU-CPP had positive impacts on their health and well-being as a result of: (a) improved diabetes self-management behaviours: *“make more proactive steps”* and (b) added connection to health and social support services *“that could help me”.*

Improved diabetes self-management behaviours: *“make more proactive steps”*. Older adults felt that the ACHRU-CPP helped them to take more action in preventing hypoglycemia and hyperglycemia and decreasing their blood pressure. They reported that providers helped them to be alert to complications that can arise from poor diabetes care.

> *What [the nurse] and [dietitian] caused me to be concerned about is to make more proactive*

For peer review only

For peer review only - http://bmjopen.bmj.com/site/about/guidelines.xhtml

> *steps, to watch out for those low blood sugars. I really didn't realize how badly they could affect you. Shaking and double vision is one thing but not being able to drive, that's quite another thing.* (OA_2_242)

Older adults indicated that they gained nutritional knowledge by participating in the ACHRU-CPP. "*I improved it [eating habits]. I had to eat more fruit and vegetables...and after that, I had to hydrate myself more and add more fibre to my diet"* (OA_5_027). Older adults perceived that changes made to their diet could lead to multiple benefits including weight loss and decreased sugar levels. "*...drinking more water and diet, I think that's what was important, and I lost weight at the same time. By eating well, fewer treats, being more careful, the sugar levels were lower"* (OA_6_005).

Older adults perceived they were able to build more muscle mass and lose weight and experienced less difficulty in climbing stairs. Some older adults felt that they were not exercising enough prior to participating in the ACHRU-CPP and perceived that the providers helped them to meet their activity goals.

> *They really helped me with the exercise piece. I had poor balance. It was the [nurse and dietitian] that really said 'why don't you try doing this? I'll give you a call this week and see if you got out to do your walk', and then I'd promise them that I would start journaling my steps so little by little I started increasing my exercise.* (OA_2_247)

Added connection to health and social support services *"that could help me".* Older adults indicated that they were referred to and connected with health and social support services (e.g., food bank, exercise program, smoking cessation, home care, social work, arts program). Some older adults required supports to meet their basic needs, as they were not able to afford groceries or travel far distances for groceries and medications. "*She [nurse] referred me [for medication delivery]*" (OA_1_061). Some older adults required mental health support to enhance their ability to manage diabetes and other chronic conditions. The intervention team followed-up with older adults after making referrals to ensure that they were connected. Older adults were

For peer review only - http://bmjopen.bmj.com/site/about/guidelines.xhtml

referred to local community resources that offered free or low-cost services. "*They told me I could go to [name of community centre] to do exercise*" (OA_4_016). Prior to participating in the ACHRU-CPP older adults indicated that they had seldom been referred to programs outside of the clinic and therefore they were not aware of available community resources.

Older adults found it important to be aware of publicly-funded resources, such as tax rebates and housing options, in case they or their loved one required these in the future. By attending group wellness sessions, they learned about the types of programs the community partner sites had to offer. *"They [community partner site] have virtual classes and they're all free. They have special classes just for seniors"* (OA_2_013).

Discussion

Key findings of this study were that the ACHRU-CPP increased in-depth dialogue with '*professional friends'* and provided person-centred care and ongoing contact with providers to prevent feelings of being alone. Group interactions brought together participants with the same type of health issues and provided peer motivation. Participants identified that the program would benefit from adaptations to address cultural and language differences among older adults living with diabetes and other conditions in Canada.

A novel finding of this study was that the ACHRU-CPP was perceived by older adults from three Canadian provinces to positively impact their self-management practices of diabetes and MCC by helping to address their broad health and social needs. This has not been documented before in similar studies [19, 20]. This may be because the ACHRU-CPP was longer and more person-centred compared to other interventions. Interventionists were able to directly assess the home context and understand the impacts of social determinants of health. Mental health concerns and lack of support can impact the ability of older adults to effectively manage

For peer review only - http://bmjopen.bmj.com/site/about/guidelines.xhtml

diabetes and lead to severe hypoglycemia, elevated HbA1c levels, a greater number of missed insulin doses, and a higher risk for diabetic ketoacidosis and mortality [36, 37, 3].

When healthcare providers recommend lifestyle changes, they need to recognize that social determinants of health such as housing, food security, social relationships, and financial stability have an impact on older adults' abilities to enact them [38]. In the current study, the intervention team assessed the social determinants of health and found ways to address them, such as by linking older adults with relevant community resources, to help overcome barriers to self-management. The intervention team targeted health literacy of older adults and caregivers through education, capacity building, and opportunities for dialogue among peers and experts. Community-based interventions were found to be most effective for Type 2 diabetes self-management compared to other interventions [21].

In the current study, older adults appreciated receiving person-centred care supported by a team of providers from primary care and community sectors and the engagement of providers outside of the intervention team (such as social workers). Due to the complex nature of diabetes and MCC, interprofessional collaboration has been found to lead to positive outcomes for persons with Type 2 diabetes, such as improvements in HbA1c levels, regular testing of blood glucose levels, and smoking cessation [39, 40].

What is unique about this study is the partnership between healthcare providers and a Program Coordinator from a local community partner site. These health and social services can be underutilized if healthcare providers are not aware of them. As per the Quintuple Aim [23] there is a need to optimize the use of existing community-based services for patients, address any barriers to accessing these services, and for strong coordination of services [41].

For peer review only

For peer review only - http://bmjopen.bmj.com/site/about/guidelines.xhtml

Person-centred care was perceived to be key strength of the ACHRU-CPP that enabled older adults to improve self-management practices related to diabetes and MCC. In working towards a person-centred learning health system, defined as a health system that integrates internal data, patient experience, and research evidence [42], the priorities and experiences of older adults should be regularly reported in data systems so that services that meet their needs are developed and evaluated as part of continuous quality improvement processes [38]. As seen in this study, patient experience can be improved by having a provider connect patients with other interdisciplinary health and social care providers to ensure that smooth transitions between services occur [43].

In practice and policy, there is a need for integrated care delivery models that leverage community partnerships to help fill gaps in meeting the complex health and social needs of older adults with diabetes. To advance Quintuple Aim outcomes [23], it is critical to assess patient experiences with receiving healthcare services as part of intervention research and practice to improve health system performance. The strengths of the study include its rigorous qualitative design and large sample size, and the inclusion of diverse participants with regards to sex, marital status, and annual income from multiple sites across Canada. A limitation of the study is the lack of cultural diversity and under-representation of older adults from marginalized communities. A Diabetes Canada roundtable of key stakeholders recently emphasized the need to implement community-based interventions [44], such as the ACHRU-CPP, to better support older adults with diabetes and MCC living in marginalized communities. The need for community-based interventions is based on the premise that racial and socioeconomic disparities disproportionally affect them and put them at an increased risk for diabetes complications and mortality [45].

For peer review only - http://bmjopen.bmj.com/site/about/guidelines.xhtml

Conclusion

Overall, older adults with diabetes and MCC reported a positive experience and felt that the ACHRU-CPP had a positive impact in supporting diabetes self-management. Study findings reveal the need to ensure that older adults receive ongoing support and contact with a collaborative team of primary care and community providers to better meet the complex needs associated with daily self-management of diabetes and MCC. Results also shine light on the broader social context that constitutes the life world of older adults and how chronic disease self-management interventions need to address these contexts comprehensively through tailoring to individual circumstances. It is our hope that these findings will help usher in a new era of contextually informed person-centred care.

For peer review only - http://bmjopen.bmj.com/site/about/guidelines.xhtml

BMJ Open

Acknowledgements

We thank the older adults and caregivers who participated in this study, as well as the nurses, dietitians, nutritionists and community program coordinators who provided the intervention. We also thank the managers of intervention teams, the recruiters, research assistants, and the study sites for their support of this study. Thanks to Robyn Connors for conducting interviews with study participants. We thank the research team in the Aging, Community and Health Research Unit (https://achru.mcmaster.ca/) for supporting this study. We would also like to thank the Community Advisory Board members, including people with lived experience with diabetes, who supported local implementation of the project in each province.

We acknowledge the following authors who are members of the **ACHRU-CPP Research Team**: **Jenny Ploeg**, School of Nursing, Faculty of Health Sciences, McMaster University, Hamilton, Ontario, Canada; **Maureen Markle-Reid**, School of Nursing, Faculty of Health Sciences, McMaster University, Hamilton, Ontario, Canada; **Ruta Valaitis**, School of Nursing, Faculty of Health Sciences, McMaster University, Hamilton, Ontario, Canada; **Kathryn Fisher,** School of Nursing, Aging, Community and Health Research Unit, Faculty of Health Sciences, McMaster University, Hamilton, Ontario, Canada; **Rebecca Ganann**, School of Nursing, Aging, Community and Health Research Unit, Faculty of Health Sciences, McMaster University, Hamilton, Ontario, Canada; **Johanne Blais**, Department of Family Medicine and Emergency Medicine, Faculty of Medicine, Université Laval, Quebec City, Quebec; **Andrea Gruneir**, Department of Family Medicine Research Program, University of Alberta, Edmonton, Alberta, Canada; **France Légaré**, VITAM-Centre de recherche en santé durable, Université Laval, Quebec City, Quebec, Canada; **Janet MacIntyre**, Faculty of Nursing, University of Prince Edward Island, Charlottetown, Prince Edward Island, Canada; **William Montelpare**,

For peer review only - http://bmjopen.bmj.com/site/about/guidelines.xhtml

494 Department of Applied Human Sciences, Faculty of Science, University of Prince Edward
495 Island, Prince Edward Island, Canada; **Jean-Sébastien Paquette**, Department of Family
496 Medicine and Emergency Medicine, Faculty of Medicine Université Laval, Québec, Canada;
497 **Marie-Eve Poitras**, Department of Family Medicine and Emergency Medicine, Faculty of
498 Medicine and Health Sciences, Université de Sherbrooke Chicoutimi, Quebec, Canada; **Angela**
499 **Riveroll**, Department of Applied Human Sciences, Faculty of Science, University of Prince
500 Edward Island, Charlottetown, Prince Edward Island, Canada, **Ali Ben Charif,** CubecXpert,
501 Quebec City, Quebec, Canada; **Dean Eurich**, School of Public Health, University of Alberta,
502 Edmonton, Alberta, Canada; **Amiram Gafni**, Department of Health Research Methods,
503 Evidence and Impact, Faculty of Health Sciences, McMaster University, Hamilton, Ontario,
504 Canada; **Gary Lewis**, Department of Medicine and Department of Physiology, University of
505 Toronto, Toronto, Ontario, Canada; **Lynne Mansell**, Patient/Public Research Partner, Alberta,
506 Canada; **Melissa Northwood**, School of Nursing, Aging, Community and Health Research Unit,
507 Faculty of Health Sciences, McMaster University, Hamilton, Ontario, Canada; **Janet Pritchard**,
508 Interdisciplinary Science and Kinesiology, Faculty of Science, McMaster University, Hamilton,
509 Ontario, Canada; **Cheryl Sadowski**, Faculty of Pharmacy and Pharmaceutical Sciences,
510 University of Alberta, Edmonton, Alberta, Canada; **Diana Sherifali**, School of Nursing, Faculty
511 of Health Sciences, McMaster University, Hamilton, Ontario, Canada; **Frank Tang**,
512 Patient/Public Research Partner, Ontario, Canada**; Lehana Thabane,** Department of Health
513 Research Methods, Evidence and Impact, Faculty of Health Sciences, McMaster University,
514 Hamilton, Ontario, Canada; **Ross Upshur**, Bridgepoint Active Healthcare, Toronto, Ontario,
515 Canada; **Tyler Williamson**, Centre for Health Informatics, Cumming School of Medicine and
516 Department of Community Health Sciences, University of Calgary, Calgary, Alberta, Canada;

For peer review only - http://bmjopen.bmj.com/site/about/guidelines.xhtml

Marie-Lee Yous, School of Nursing, Faculty of Health Sciences, McMaster University, Hamilton, Ontario, Canada.

Author Contributions

Conceptualization: JP, MMR, RV, KF, RG, FL, WM.

Formal Analysis: MLY, RG, TC, JP.

Funding Acquisition: JP, MMR, RV, KF, RG, AG, FL, JM, WM.

Investigation: JP, MMR, RV, KF, RG, TC, FL, WM, MLY.

Methodology: JP, MMR, RV, KF, RG, MLY, FL, WM.

Project Administration: JP, MMR, RV, TC, FL, WM.

Resources: JP, MMR, RV, FL, WM.

Supervision: JP, MMR, RV, RG, TC, FL, WM.

Validation: MLY, JP, MMR, RV, KF, RG, TC, RC.

Writing – Original Draft: MLY.

Writing – Review and Editing: MLY, RG, TC, JP, MMR, RV, KF, FL, JM, WM. The authors read and approved the final manuscript.

Funding

This study is supported, in part, by funding from the Canadian Institutes of Health Research Strategy for Patient-Oriented Research (SPOR) Primary and Integrated Health Care Innovations Network: Programmatic Grants (Funding Reference Number: KPG-156883) in partnership with: Diabetes Action Canada, a Canadian Institutes for Health Research (CIHR) Strategy for Patient-Oriented Research Network in Chronic Disease (project reference #1.1.1ACHR); McMaster Institute for Research on Aging (Hamilton, ON); McMaster University School of Nursing; Réseau-1 Québec; Fonds de Recherche du Québec (FRQS); Scarborough

For peer review only - http://bmjopen.bmj.com/site/about/guidelines.xhtml

BMJ Open

Health Network Foundation. This research was also undertaken, in part, thanks to the funding from Dr. Markle-Reid's Tier 2 CIHR Canada Research Chair. The study was investigator-initiated. The funders of this study had no role in study design, data collection, data analysis, data interpretation or writing the manuscript.

Competing Interests

None declared.

Availability of Data and Materials

The data for this research consists of questionnaires and interviews. Raw data cannot be publicly released due to the risk of compromising participant confidentiality

Ethics Statement

Institutional ethics approval was obtained from the following: the Hamilton Integrated Research Ethics Board (#5101); the Scarborough Health Network Research Ethics Board (#NEP-18-014); the Unity Health Toronto Research Ethics Board (#18-336); University of Prince Edward Island Research Ethics Board (#6008019); Prince Edward Island Research Ethics Board (#6008019); and Centre intégré universitaire de santé et de services sociaux (CIUSSS) de la Capitale-Nationale (MP-13-2019-1670). Trained research assistants obtained informed consent from all older adult participants prior to their participation in the study, and all participants received a copy of their consent form, in person or by mail. All participants gave permission to audio-record their interview. All participant information was kept confidential in a secure location (e.g., locked cabinet in a secured office, password-protected encrypted electronic folders), and data were de-identified using unique study IDs. Participants who completed a qualitative interview received a $25.00 gift card as an honorarium.

For peer review only - http://bmjopen.bmj.com/site/about/guidelines.xhtml

References

[1] World Health Organization. Diabetes. Available from: https://www.who.int/en/news-room/fact-sheets/detail/diabetes. 2022. Accessed 3 Jan 2022.

[2] Kirkman MS, Briscoe VJ, Clark N, et al. Diabetes in older adults: A consensus report. *J Am Geriatr Soc* 2012;35(12):2650-64.

[3] Dhaliwal R, Weinstock RS. Management of type 1 diabetes in older adults. *Diabetes Spectr*. 2014;27(1):9-20.

[4] Sinnige J, Braspenning J, Schellevis F, et al. The prevalence of disease clusters in older adults with multiple chronic diseases–a systematic literature review. *PloS one* 2013;8(11):e79641.

[5] Lin PJ, Kent DM, Winn A, et al. Multiple chronic conditions in type 2 diabetes mellitus: prevalence and consequences. *Am J Manag Care* 2015;21(1):e23-34.

[6] Fisher K, Griffith L, Gruneir A, et al. Comorbidity and its relationship with health service use and cost in community-living older adults with diabetes: A population-based study in Ontario, Canada. *Diabetes Res Clin Pract* 2016;122:113-123.

[7] Willi C, Bodenmann P, Ghali WA, et al. Active smoking and the risk of type 2 diabetes: A systematic review and meta-analysis. *JAMA* 2007; 298:2654-64.

[8] Ploeg J, Matthew-Maich N, Fraser K, et al. Managing multiple chronic conditions in the community: A Canadian qualitative study of the experiences of older adults, family caregivers and healthcare providers. *BMC Geriatr* 2017;17.

[9] The Change Foundation. Family caregiver assessment in health care settings, summary of the change foundation's literature review and environment scan project. Toronto, August 2016.

[10] McGilton KS, Vellani S, Yeung L, et al. Identifying and understanding the health and social care needs of older adults with multiple chronic conditions and their caregivers: A scoping review. BMC Geriatr 2018;18(1):1-33.

[11Craig P, Dieppe P, Macintyre S, et al. Developing and evaluating complex interventions: the new Medical Research Council guidance. *BMJ*. 2008;337:1-6.

[12] Whittemore R. A systematic review of the translational research on the diabetes prevention program. *Transl Behav Med* 2011;1:480-91.

[13] Tuomilehto J, Schwarz P, Lindström J. Long-term benefits from lifestyle interventions for type 2 diabetes prevention: Time to expand the efforts. *Diabetes Care* 2011;34:S210-14.

[14] Smith SM, Wallace E, O'Dowd T, et al. Interventions for improving outcomes in patients with multimorbidity in primary care and community settings. *Cochrane Database Syst Rev* 2016;3.

[15] Yoon U, Kwok LL, Magkidis A. Efficacy of lifestyle interventions in reducing diabetes incidence in patients with impaired glucose tolerance: A systematic review of randomized controlled trials. *Metabolism* 2013;62:303-14.

[16] Busetto L, Luijkx KG, Elissen AMJ, et al. Context, mechanisms and outcomes of integrated care for diabetes mellitus type 2: A systematic review. *BMC Health Serv Res* 2016;16:1-14.

[17] Diabetes Prevention Program Research Group. 10-year follow-up of diabetes incidence and weight loss in the diabetes prevention program outcomes study. *Lancet* 2009;374:1677-86.

[18] Wong KC, Wong FKY, Yeung W, et al. The effect of complex interventions on supporting self-care among community-dwelling older adults: A systematic review and meta-analysis. *Age Ageing* 2017;1-9.

For peer review only

For peer review only - http://bmjopen.bmj.com/site/about/guidelines.xhtml

[19] Azami G, Soh KL, Sazlina SG, et al. Effect of a nurse-led diabetes self-management education program on glycosylated hemoglobin among adults with type 2 diabetes. *J Diabetes Res* 2018:1-13.
[20] Chow SK, Wong FK. A randomized controlled trial of a nurse-led case management programme for hospital-discharged older adults with co-morbidities. *J Adv Nurs* 2014;70(10):2257-71.
[21] Dahal PK, Hosseinzadeh H. Association of health literacy and diabetes self-management: A systematic review. *Aust J Prim Health* 2020;25(6):526-33.
[22] Ferris R, Blaum C, Kiwak E, et al. Perspectives of patients, clinicians, and health system leaders on changes needed to improve the health care and outcomes of older adults with multiple chronic conditions. *J Aging Health* 2018;30(5):778-99.
[23] Nundy S, Cooper LA, Mate KS. The Quintuple Aim for health care improvement: A new imperative to advance health equity. *JAMA* 2022;327(6):521–522.
[24] Markle-Reid M, Ploeg J, Fisher K, et al. The Aging, Community and Health Research Unit—Community Partnership Program for older adults with type 2 diabetes and multiple chronic conditions: A feasibility study. *Pilot Feasibility Stud* 2016 2(1):24.
[25] Markle-Reid M, Ploeg J, Fraser KD, et al. Community program improves quality of life and self-management in older adults with diabetes mellitus and comorbidity. J Am Geriatr Soc. 2018 Feb;66(2):263-73.
[26] Miklavcic JJ, Fraser KD, Ploeg J, Markle-Reid M, Fisher K, Gafni A, Griffith LE, Hirst S, Sadowski CA, Thabane L, Triscott JA. Effectiveness of a community program for older adults with type 2 diabetes and multimorbidity: A pragmatic randomized controlled trial. BMC Geriatr. 2020 Dec;20(1):1-14.
[27] Fisher K, Markle-Reid M, Ploeg J, Bartholomew A, Griffith LE, Gafni A, Thabane L, Yous ML. Self-management program versus usual care for community-dwelling older adults with multimorbidity: A pragmatic randomized controlled trial in Ontario, Canada. J Comorb. 2020 Oct 14;20(174):1-14.
[28] Ploeg J, Markle-Reid M, Valaitis R, et al. The Aging, Community and Health Research Unit Community Partnership Program for older adults with diabetes and multiple chronic conditions: Study protocol for a randomized controlled trial. *BMC Geriatrics* 2022;22(99):1-22.
[29] Sandelowski M. Whatever happened to qualitative description? Res Nurs health. 2000;23(4):334-40.
[30] Sandelowski M. What's in a name? Qualitative description revisited. Res Nurs Health. 2010 Feb;33(1):77-84.
[31] Patton MQ. Qualitative evaluation and research methods. Beverly Hills, CA: SAGE, 1990.
[32] Braun V, Clarke V. Successful qualitative research: A practical guide for beginners. London, England: SAGE, 2013.
[33] Damschroder LJ, Aron DC, Keith RE, et al. Fostering implementation of health services research findings into practice: A consolidated framework for advancing implementation science. Implement Sci. 2009 Dec;4(1):1-5.
[34] QSR International Pty Ltd. NVivo (Version 12). 2018. Available from: https://www.qsrinternational.com/nvivo-qualitative-data-analysis-software/home
[35] Lincoln YS, Guba EG. Naturalistic inquiry. Sage Publications, 1985.
[36] Robinson DJ, Coons M, Haensel H, et al. Diabetes and mental health. *Can J Diabetes* 2018 Apr 1;42:S130-41.

For peer review only - http://bmjopen.bmj.com/site/about/guidelines.xhtml

[37] Lynch CP, Gebregziabher M, Zhao Y, et al. Impact of medical and psychiatric multi-morbidity on mortality in diabetes: emerging evidence. *BMC Endocr Disord* 2014;14(1):1-8.
[38] Kuluski K, Guilcher SJ. Toward a person-centred learning health system: Understanding value from the perspectives of patients and caregivers. *Healthc Pap* 2019;18(4):36-46.
[39] Hellquist K, Bradley R, Grambart S, et al. Collaborative practice benefits patients: An examination of interprofessional approaches to diabetes care. *Health Interprofessional Pract* 2012;1(eP1017).
[40] O'Connor PJ, Desai J, Solberg LI, et al. Randomized trial of quality improvement intervention to improve diabetes care in primary care settings. *Diabetes Care* 2005;28,1890–97.
[41] Valaitis RK, Wong ST, MacDonald M, et al. Addressing quadruple aims through primary care and public health collaboration: ten Canadian case studies. BMC Public Health 2020;20(1):1-6.
[42] Agency for Healthcare Research and Quality. About learning health systems. 2019. Available from: https://www.ahrq.gov/learning-health-systems/about.html. Accessed 12 Feb 2022.
[43] Rapid Improvement Support and Exchange. RISE brief 1. OHT (Ontario Health Team) building blocks. 2019. Available from: https://www.mcmasterforum.org/docs/default-source/rise-docs/rise-briefs/rb1_oht-building-blocks.pdf?sfvrsn=71b154d5_27. Accessed 12 Feb 2022.
[44] Diabetes Canada. Summary of Diabetes Canada Diabetes 360˚ Ontario roundtable. 2019. Available from: https://www.diabetes.ca/DiabetesCanadaWebsite/media/Advocacy-and-Policy/Advocacy%20Reports/Ontario-Diabetes-360-Strategy_Roundtable-Summary_FINAL.pdf. Accessed 12 Feb 2022.
[45] Clements JM, West BT, Yaker Z, et al. Disparities in diabetes-related multiple chronic conditions and mortality: The influence of race. *Diabetes Res Clin Pract* 2020;159(107984):1-19.

For peer review only - http://bmjopen.bmj.com/site/about/guidelines.xhtml

COREQ (COnsolidated criteria for REporting Qualitative research) Checklist

A checklist of items that should be included in reports of qualitative research. You must report the page number in your manuscript where you consider each of the items listed in this checklist. If you have not included this information, either revise your manuscript accordingly before submitting or note N/A.

Topic	Item No.	Guide Questions/Description	Reported on Page No.
**Domain 1: Research team and reflexivity**			
Personal characteristics			
Interviewer/facilitator	1	Which author/s conducted the interview or focus group?	
Credentials	2	What were the researcher's credentials? E.g. PhD, MD	
Occupation	3	What was their occupation at the time of the study?	
Gender	4	Was the researcher male or female?	
Experience and training	5	What experience or training did the researcher have?	
Relationship with participants			
Relationship established	6	Was a relationship established prior to study commencement?	
Participant knowledge of the interviewer	7	What did the participants know about the researcher? e.g. personal goals, reasons for doing the research	
Interviewer characteristics	8	What characteristics were reported about the inter viewer/facilitator? e.g. Bias, assumptions, reasons and interests in the research topic	
**Domain 2: Study design**			
Theoretical framework			
Methodological orientation and Theory	9	What methodological orientation was stated to underpin the study? e.g. grounded theory, discourse analysis, ethnography, phenomenology, content analysis	
Participant selection			
Sampling	10	How were participants selected? e.g. purposive, convenience, consecutive, snowball	
Method of approach	11	How were participants approached? e.g. face-to-face, telephone, mail, email	
Sample size	12	How many participants were in the study?	
Non-participation	13	How many people refused to participate or dropped out? Reasons?	
Setting			
Setting of data collection	14	Where was the data collected? e.g. home, clinic, workplace	
Presence of non-participants	15	Was anyone else present besides the participants and researchers?	
Description of sample	16	What are the important characteristics of the sample? e.g. demographic data, date	
Data collection			
Interview guide	17	Were questions, prompts, guides provided by the authors? Was it pilot tested?	
Repeat interviews	18	Were repeat inter views carried out? If yes, how many?	
Audio/visual recording	19	Did the research use audio or visual recording to collect the data?	
Field notes	20	Were field notes made during and/or after the inter view or focus group?	
Duration	21	What was the duration of the inter views or focus group?	
Data saturation	22	Was data saturation discussed?	
Transcripts returned	23	Were transcripts returned to participants for comment and/or	

For peer review only

Topic	Item No.	Guide Questions/Description	Reported on Page No.
		correction?	
**Domain 3: analysis and findings**			
Data analysis			
Number of data coders	24	How many data coders coded the data?	
Description of the coding tree	25	Did authors provide a description of the coding tree?	
Derivation of themes	26	Were themes identified in advance or derived from the data?	
Software	27	What software, if applicable, was used to manage the data?	
Participant checking	28	Did participants provide feedback on the findings?	
Reporting			
Quotations presented	29	Were participant quotations presented to illustrate the themes/findings? Was each quotation identified? e.g. participant number	
Data and findings consistent	30	Was there consistency between the data presented and the findings?	
Clarity of major themes	31	Were major themes clearly presented in the findings?	
Clarity of minor themes	32	Is there a description of diverse cases or discussion of minor themes?	

Developed from: Tong A, Sainsbury P, Craig J. Consolidated criteria for reporting qualitative research (COREQ): a 32-item checklist for interviews and focus groups. *International Journal for Quality in Health Care*. 2007. Volume 19, Number 6: pp. 349 – 357

Once you have completed this checklist, please save a copy and upload it as part of your submission. DO NOT include this checklist as part of the main manuscript document. It must be uploaded as a separate file.

For peer review only

BMJ Open

Older Adults' Experiences and Perceived Impacts of the Aging, Community and Health Research Unit – Community Partnership Program (ACHRU-CPP) for Diabetes Self-Management in Canada: A Qualitative Descriptive Study

Journal:	*BMJ Open*
Manuscript ID	bmjopen-2022-068694.R1
Article Type:	Original research
Date Submitted by the Author:	11-Feb-2023
Complete List of Authors:	Yous, Marie-Lee; McMaster University Faculty of Health Sciences, School of Nursing<br>Ganann, Rebecca; McMaster University Faculty of Health Sciences, School of Nursing, Aging, Community and Health Research Unit<br>Ploeg, Jenny ; McMaster University Faculty of Health Sciences, School of Nursing, Aging, Community and Health Research Unit<br>Markle-Reid, Maureen; McMaster University Faculty of Health Sciences, School of Nursing, Aging, Community and Health Research Unit<br>Northwood, Melissa ; McMaster University Faculty of Health Sciences, School of Nursing, Aging, Community and Health Research Unit<br>Fisher, Kathryn ; McMaster University School of Nursing, School of Nursing, Aging, Community and Health Research Unit<br>Valaitis, Ruta; McMaster University Faculty of Health Sciences, School of Nursing, Aging, Community and Health Research Unit<br>Chambers, Tracey; McMaster University Faculty of Health Sciences, School of Nursing, Aging, Community and Health Research Unit<br>Montelpare, William; University of Prince Edward Island, Department of Applied Human Sciences, Faculty of Science<br>Légaré, France; VITAM Centre de recherche en santé durable; Laval University, Department of Family and Emergency Medicine, Faculty of Medicine<br>Beleno, Ron; McMaster University Faculty of Health Sciences, School of Nursing, Aging, Community and Health Research Unit<br>Gaudet, Gary; University of Prince Edward Island<br>Giacometti, Luisa; McMaster University Faculty of Health Sciences, School of Nursing, Aging, Community and Health Research Unit<br>Levely, Deborah; McMaster University Faculty of Health Sciences, School of Nursing, Aging, Community and Health Research Unit<br>Lindsay, Craig; McMaster University Faculty of Health Sciences, School of Nursing, Aging, Community and Health Research Unit<br>Morrison, Allan; University of Prince Edward Island<br>Tang, Frank; McMaster University Faculty of Health Sciences, School of Nursing, Aging, Community and Health Research Unit<br>ACHRU-CPP Research Team, on behalf of the; McMaster University Faculty of Health Sciences, School of Nursing
**Primary Subject Heading**:	Diabetes and endocrinology

Secondary Subject Heading:	Qualitative research
Keywords:	QUALITATIVE RESEARCH, PRIMARY CARE, DIABETES & ENDOCRINOLOGY

SCHOLARONE™
Manuscripts

I, the Submitting Author has the right to grant and does grant on behalf of all authors of the Work (as defined in the below author licence), an exclusive licence and/or a non-exclusive licence for contributions from authors who are: i) UK Crown employees; ii) where BMJ has agreed a CC-BY licence shall apply, and/or iii) in accordance with the terms applicable for US Federal Government officers or employees acting as part of their official duties; on a worldwide, perpetual, irrevocable, royalty-free basis to BMJ Publishing Group Ltd ("BMJ") its licensees and where the relevant Journal is co-owned by BMJ to the co-owners of the Journal, to publish the Work in this journal and any other BMJ products and to exploit all rights, as set out in our licence.

The Submitting Author accepts and understands that any supply made under these terms is made by BMJ to the Submitting Author unless you are acting as an employee on behalf of your employer or a postgraduate student of an affiliated institution which is paying any applicable article publishing charge ("APC") for Open Access articles. Where the Submitting Author wishes to make the Work available on an Open Access basis (and intends to pay the relevant APC), the terms of reuse of such Open Access shall be governed by a Creative Commons licence – details of these licences and which Creative Commons licence will apply to this Work are set out in our licence referred to above.

Other than as permitted in any relevant BMJ Author's Self Archiving Policies, I confirm this Work has not been accepted for publication elsewhere, is not being considered for publication elsewhere and does not duplicate material already published. I confirm all authors consent to publication of this Work and authorise the granting of this licence.

For peer review only

BMJ Open

Original Research

Title: Older Adults' Experiences and Perceived Impacts of the Aging, Community and Health Research Unit – Community Partnership Program (ACHRU-CPP) for Diabetes Self-Management in Canada: A Qualitative Descriptive Study

Corresponding Author: Marie-Lee Yous, School of Nursing, Aging, Community and Health Research Unit, Faculty of Health Sciences, McMaster University, 1280 Main Street West, Room HSC 3N25, Hamilton, Ontario L8S 4K1, Canada, Email: yousm@mcmaster.ca; ORCID: 0000-0002-4271-0401

Marie-Lee Yous, School of Nursing, Aging, Community and Health Research Unit, Faculty of Health Sciences, McMaster University, Hamilton, Ontario, Canada, email: yousm@mcmaster.ca ORCID: 0000-0002-4271-0401

Rebecca Ganann, School of Nursing, Aging, Community and Health Research Unit, Faculty of Health Sciences, McMaster University, email: ganannrl@mcmaster.ca ORCID: 0000-0002-7566-8932

Jenny Ploeg, School of Nursing, Aging, Community and Health Research Unit, Faculty of Health Sciences, McMaster University, Hamilton, Ontario, Canada, email: ploegj@mcmaster.ca ORCID: 0000-0001-8168-8449

Maureen Markle-Reid, School of Nursing, Aging, Community and Health Research Unit, Faculty of Health Sciences, McMaster University, Hamilton, Ontario, Canada, email: mreid@mcmaster.ca ORCID: 0000-0002-4019-7077

Melissa Northwood, School of Nursing, Aging, Community and Health Research Unit, Faculty of Health Sciences, McMaster University, Hamilton, Ontario, Canada, email: northwm@mcmaster.ca ORCID: 0000-0001-5043-8068

Kathryn Fisher, School of Nursing, Aging, Community and Health Research Unit, Faculty of Health Sciences, McMaster University, Hamilton, Ontario, Canada fisheka@mcmaster.ca ORCID: 0000-0001-8342-1238

Ruta Valaitis, School of Nursing, Aging, Community and Health Research Unit, Faculty of Health Sciences, McMaster University, Hamilton, Ontario, Canada, email: valaitis@mcmaster.ca ORCID: 0000-0002-3117-0542

Tracey Chambers, School of Nursing, Aging, Community and Health Research Unit, Faculty of Health Sciences, McMaster University, Hamilton, Ontario, Canada, email: chambt@mcmaster.ca ORCID: 0000-0002-9325-6894

William Montelpare, Margaret and Wallace McCain Chair in Human Development and Health, Department of Applied Human Sciences, Faculty of Science, University of Prince Edward

For peer review only - http://bmjopen.bmj.com/site/about/guidelines.xhtml

Island, Charlottetown, Prince Edward Island, Canada, email: wmontelpare@upei.ca ORCID: 0000-0002-4167-4613

France Légaré, VITAM-Centre de recherche en santé durable, Université Laval, Québec City, Québec, Canada, email: France.legare@fmed.ulaval.ca ORCID: 0000-0002-2296-6696

Ron Beleno, Patient/Public Research Partner, Aging, Community and Health Research Unit, Faculty of Health Sciences, McMaster University, Hamilton, Ontario, Canada email: ron@rb33.com

Gary Gaudet, Patient/Public Research Partner, University of Prince Edward Island, Charlottetown, Prince Edward Island, Canada, email: l.ggaudet@bellaliant.net

Luisa Giacometti, Patient/Public Research Partner, School of Nursing, Aging, Community and Health Research Unit, Faculty of Health Sciences, McMaster University, Hamilton, Ontario, Canada, email: luisag@bell.net

Deborah Levely, Patient/Public Research Partner, School of Nursing, Aging, Community and Health Research Unit, Faculty of Health Sciences, McMaster University, Hamilton, Ontario, Canada, email: deborah.levely@rogers.com

Craig Lindsay, Patient/Public Research Partner, School of Nursing, Aging, Community and Health Research Unit, Faculty of Health Sciences, McMaster University, Hamilton, Ontario, Canada, email: lindsay.cr@gmail.com

Allan Morrison, Patient/Public Research Partner, University of Prince Edward Island, Charlottetown, Prince Edward Island, Canada, email: allan.morrison62.am@gmail.com

Frank Tang, Patient/Public Research Partner, School of Nursing, Aging, Community and Health Research Unit, Faculty of Health Sciences, McMaster University, Hamilton, Ontario, Canada, email: frankmtang@gmail.com

On behalf of the ACHRU-CPP Research Team

Word count excluding title page, abstract, references, figures and tables **(max 4,000)**: 4,672

For peer review only - http://bmjopen.bmj.com/site/about/guidelines.xhtml

BMJ Open

Abstract (max 300 words): 300 words

Objectives: To assess the experiences and perceived impacts of the Aging, Community and Health Research Unit – Community Partnership Program (ACHRU-CPP) from the perspectives of older adults with diabetes and other chronic conditions. The ACHRU-CPP is a complex 6-month self-management evidence-based intervention for community-living older adults aged 65 years or older with Type 1 or Type 2 diabetes and at least one other chronic condition. It includes home and phone visits, care coordination, system navigation support, caregiver support, and group wellness sessions delivered by a nurse, dietitian or nutritionist, and community program coordinator.

Design: Qualitative descriptive design embedded within a randomized controlled trial was used.

Setting: Six trial sites offering primary care services from three Canadian provinces (i.e., Ontario, Quebec, and Prince Edward Island) were included.

Participants: The sample was 45 community-living older adults aged 65 years or older with diabetes and at least one other chronic condition.

Methods: Participants completed semi-structured post-intervention interviews by phone in English or French. The analytical process followed Braun and Clarke's experiential thematic analysis framework. Patient partners informed study design and interpretation.

Results: The mean age of older adults was 71.7 years, and the mean length of time living with diabetes was 18.8 years. Older adults reported positive experiences with the ACHRU-CPP that supported diabetes self-management such as, improved knowledge in managing diabetes and other chronic conditions, enhanced physical activity and function, improved eating habits, and opportunities for socialization. They reported being connected to community resources by the intervention team to address social determinants of health and support self-management.

For peer review only - http://bmjopen.bmj.com/site/about/guidelines.xhtml

Conclusions: Older adults perceived that a 6-month person-centred intervention collaboratively delivered by a team of health and social care providers helped support chronic disease self-management. There is a need for providers to help older adults connect with available health and social services in the community.

Strengths and limitations of this study

- This study included a rigorous qualitative design with a large sample size.
- A rigorous analytic method was used involving multiple researchers with expertise in primary care, qualitative, ageing, and diabetes research, as well as programme evaluation.
- Patient and public research partners were involved in designing the intervention, informing the study design and interview guides, interpreting the results, and developing the manuscript.
- A limitation of the study was related to the sample as there was a lack of cultural diversity with regards to ethnicity and under-representation of older adults from marginalized communities.

Keywords: diabetes self-management, older adults, community, primary care, qualitative

For peer review only - http://bmjopen.bmj.com/site/about/guidelines.xhtml

Introduction

As of 2022, approximately 422 million people have been diagnosed with diabetes mellitus worldwide [1]. Older adults are more likely to have Type 2 diabetes than younger adults [2] and are at risk for hypoglycemia which can adversely affect cognition, vision, hearing, mobility, and mental health [3] as well as self-care activities including exercise and diet. More than 40% of older adults with diabetes have three or more chronic conditions [4], including hyperlipidemia, hypertension, asthma, chronic obstructive pulmonary disease, chronic kidney disease, arthritis, and heart failure [5]. Following management plans for one condition may be challenging due to symptoms or conflicting guidelines from another condition. Higher burden associated with the presence of multiple chronic conditions (MCC) has been linked to higher risk for mortality, decreased physical and mental functioning, and increased health services use [2, 6, 7]. Community-dwelling older adults with MCC are highly reliant on family/friend caregivers for support [8], which can lead to poor mental and physical health, and financial losses among caregivers [9]. Caregivers' unmet needs [10] can lead to their increased use of hospital and emergency services.

Complex health interventions are defined as having multiple interacting components [11]. For older adults with diabetes, complex health interventions such as peer support programs, have demonstrated positive effects in managing their complex needs, sustaining lifestyle changes, and achieving health benefits [12-17]. Complex interventions that target self-care and incorporate opportunities for peer-to-peer discussions among community-dwelling older adults can improve their mental and physical health and reduce falls [18, 19]. Nurse-led self-management programs for diabetes and other chronic conditions can lead to improvements in self-rated health, glycated hemoglobin (HbA1c) values, blood pressure, weight, and self-management behaviours [19, 20].

For peer review only

For peer review only - http://bmjopen.bmj.com/site/about/guidelines.xhtml

Providing diabetes self-management programs through partnerships between primary care and community organizations (e.g., senior centres, YMCA) supports program uptake, implementation, and sustainability [12], which can lead to improved health literacy (i.e., being able to locate, read and understand health information for informed decision-making). In a systematic review of randomized controlled trials (RCTs), health literacy was instrumental in enhancing diabetes knowledge, self-efficacy, and physical activity [21].

Receiving care from multiple providers from different health and community providers can lead to fragmented care for older adults, as referrals across organizations are often not well integrated [22]. Seamless care coordination and system navigation for older adults with MCC remain high priorities for this population. There is a need for innovative programs for older adults that focus on the Quintuple Aims of high-quality care: (a) enhancing the patient experience of care; (b) creating healthy populations; (c) reducing healthcare costs; (d) improving the care delivery experience; and (e) health equity [23]. Gaps in previous intervention designs include lack of emphasis on patient experience of care and considerations for health equity [23].

The Aging, Community and Health Research Unit – Community Partnership Program (ACHRU-CPP) is a 6-month self-management intervention for community-living older adults (≥ 65 years old), diagnosed with Type 1 or Type 2 diabetes and at least one other chronic condition, and their family/friend caregivers (≥ 18 years old). The intervention was evaluated in a feasibility study in Ontario, Canada [24], followed by a clinical trial in selected primary care and community settings in two Canadian provinces (Ontario and Alberta) [25-27]. A multi-jurisdictional pragmatic RCT is currently in progress to evaluate the implementation and effectiveness of the ACHRU-CPP in three Canadian provinces. To better understand how to

For peer review only - http://bmjopen.bmj.com/site/about/guidelines.xhtml

BMJ Open

address the complex needs of older adults, we sought to assess the experiences and perceived impacts of the ACHRU-CPP from the perspectives of older adults with diabetes and MCC.

Methods

Design

This qualitative study is embedded within the multi-site implementation-effectiveness type II hybrid RCT, as outlined in the protocol paper [28]. This study used a qualitative descriptive design, as described by Sandelowski [29, 30], to provide a fulsome summary while remaining close to the words of participants when describing their experiences with the ACHRU-CPP and its perceived impacts.

ACHRU-CPP Program

The ACHRU-CPP is delivered by an interprofessional team of primary care providers, which includes a Registered Nurse (RN) and Registered Dietitian (RD) or Nutritionist from a primary care setting, and a Program Coordinator (e.g., Registered Kinesiologist) from a local community partner organization (hereafter referred to as the intervention team). Table 1 summarizes the core components of the ACHRU-CPP. Due to the COVID-19 pandemic some participants received virtual visits by phone or videoconferencing. A comparison of results from the virtual and in-person approaches will be published in a future paper.

For peer review only

Table 1. The ACHRU-CPP five core components

Intervention Components	Goals
1. Home/virtual visits (up to 3 home visits) and unlimited follow-up phone calls by a RN and/or RD or Nutritionist	To assess older adults' and caregivers' needs and goals using standardized tools to support a coordinated care plan
2. Monthly group wellness sessions (up to 6 sessions) at a local community centre led by	To provide older adults and caregivers with gentle progressive physical activity, self-

For peer review only - http://bmjopen.bmj.com/site/about/guidelines.xhtml

BMJ Open

Intervention Components	Goals
the RN, RD or Nutritionist and Community Program Coordinator	management education for diabetes and other chronic conditions, and healthy lunches and snacks
3. Monthly team case conferences which include a RN, RD or Nutritionist, and Community Program Coordinator	To discuss the health and social care needs of older adults and caregivers, develop and revise the coordinated care plan, and plan topics for group wellness sessions
4. Collaboration with the primary care interprofessional team and other specialists (e.g., family physicians, nurse practitioners, kinesiologists, social workers, home care and social service providers, pharmacists, endocrinologists)	To support primary care and community providers in working collaboratively to develop care plans for older adults, and connect older adults and caregivers to specialists and community resources
5. Nurse-led care coordination/system navigation	To facilitate linkages to other primary healthcare providers, specialists and community care services for older adults and caregivers

Note. RN = Registered Nurse; RD = Registered Dietitian

Patient and Public Involvement

The need for the program was originally identified by community-dwelling older adults with diabetes and other chronic conditions and their caregivers and was subsequently co-designed by older adults in collaboration with primary and community care providers and researchers [24]. In the current RCT, patient partners from the pan-Canadian Steering Committee were involved in reviewing research questions and advising the research team on the selection of outcome measures [28]. Patient and public research partners also participated in local Community Advisory Boards in each site to inform further adaptations to the intervention and support local implementation. Patient and public research partners from the local community advisory boards (RB, GG, LG, CL, DL, AM) and the Steering Committee (LM, FT) also provided input into development of this manuscript by reviewing and interpreting the results and helping to shape the key messages.

For peer review only - http://bmjopen.bmj.com/site/about/guidelines.xhtml

Setting

The study was conducted in two sites in Ontario, Quebec, and Prince Edward Island, Canada. Each of the six sites was selected to ensure variability in geographic setting (urban and rural); socio-demographic and cultural backgrounds; language spoken (English or French); demonstrated support for the ACHRU-CPP; and the presence of staff to support intervention implementation. An RN and RD or Nutritionist from a primary care setting or diabetes education program worked in partnership with a program coordinator from a local community partner site (e.g., YMCA) to implement the program.

Sample and Recruitment

Older adults were screened for eligibility to participate in the RCT by a trained staff member of the primary care site, as described in the study protocol [28]. Eligible patients met the following inclusion criteria: (a) aged 65 years or older; (b) diagnosis of Type 1 or Type 2 diabetes with at least one other chronic condition; (c) receiving primary care services from one of the participating primary care settings; (d) living within the area served by the primary care setting and community site; (e) able to provide informed consent or has a substitute decision-maker able to provide informed consent on the patient's behalf; and (f) competent in English or French, or has an interpreter competent in English or French.

Following the completion of baseline interviews, patients were randomized to receive the intervention (i.e., ACHRU-CPP) in addition to usual care or usual care alone. A total of 8-10 older adults per site who completed the 6-month intervention were invited to participate in follow-up telephone interviews. Trained research assistants (MY, RC), with no prior relationship with participants, used a telephone script to call selected older adults within two weeks of completing the ACHRU-CPP, to invite them to participate in a telephone interview. Maximum

For peer review only - http://bmjopen.bmj.com/site/about/guidelines.xhtml

BMJ Open

variation purposive sampling [31] was used to select a diverse sample of participants across all 6 sites based on their sex, annual income, ethnicity, and level of participation in all components of the study.

Data Collection

Semi-structured post-intervention telephone interviews were conducted between April 2020 and August 2021. Trained research assistants conducted audio-recorded interviews, ranging from 20 to 60 minutes in length, in English or French. Interviews were transcribed verbatim by experienced transcriptionists. Interviews conducted in English were transcribed and cleaned by trained research staff, while interviews conducted in French were transcribed and translated into English by professional transcriptionists and later validated by a bilingual member of the research team. Transcripts were not returned to participants for their review. The interview guide was created based on: (a) a review of the literature of health and social needs of older adults and caregivers, patient-provider communication, and system navigation and (b) feedback from patient partners and the research team with expertise in aging, community-based supports for older adults and caregivers, and qualitative research. Table 2 provides sample interview questions.

Table 2. Sample interview questions for older adults

**Questions for Older Adults**
1. What did you need the most in the past six months (e.g., physical, emotional, mental or psychological support, transportation, financial assistance, housekeeping, personal care support)?
2. When [name of nurse and dietitian] visited you what types of things did they do during those visits?

For peer review only - http://bmjopen.bmj.com/site/about/guidelines.xhtml

3. For other people who are living with diabetes and other chronic conditions, would you recommend that a nurse or dietitian, such as [name of nurse and dietitian], visit the person at home, make phone calls or both?
4. What types of things did you do at the monthly wellness sessions or during the individual calls with [name of the community program coordinator]?
5. How, if at all, did [name of nurse and dietitian] involve you in decisions about your care?
6. How, if at all, did [name of nurse and dietitian] help you to connect with other community health or social services to help you?
7. To what extent did the nurse and dietitian help to address your needs or the issues that were most important to you?
8. How happy are you with the overall care that you received from [name of nurse and dietitian]?
9. Was the information given by [name of nurse and dietitian] and other health professionals about care consistent (across individuals)?
10. Is there anything else about your experiences with [name of nurse and dietitian] that you would like to add that we haven't already discussed?

Data Analysis

Themes were generated using the Braun and Clarke's experiential thematic analysis framework [32] and organized under relevant constructs of the Consolidated Framework for Implementation Research framework. [33]. Thematic analysis was selected to ensure that the development of themes was informed by the experiences and perceived intervention benefits of older adults. The six phases of thematic analysis include: (a) becoming familiar with the data; (b) coding; (c) developing themes; (d) reviewing themes; (e) constructing a definition for themes and labelling them; and (f) creating a report [32]. A female research assistant with doctoral level training in qualitative research (MY) used the data management software NVivo version 12 [34] to perform coding. MY is fluent in both languages and coded in English. Results were shared

For peer review only - http://bmjopen.bmj.com/site/about/guidelines.xhtml

with the team in English only. Following the creation of codes, these were further examined for patterns to generate themes. Themes were shared with the research team, including patient partners, to ensure they were reflective of the data.

Rigour and Trustworthiness

Consensus was reached by all authors prior to the inclusion of themes in the final report. Lincoln and Guba's validation criteria [35] were applied in this study to enhance the study's rigour. To support the credibility of findings, investigator triangulation was used in data analysis through team meetings with 5-7 members to review the coding structure and evidence of themes. These members included patient and public research partners and researchers of various disciplines with expertise in qualitative research, gerontology, and community-based interventions. Conflicts were resolved through team consensus. To facilitate transferability of findings, the study sample and setting were described in detail. To support dependability and confirmability of findings, the research team kept an audit trail of study processes.

Results

A total of 295 older adults were enrolled in the RCT and randomly allocated to receive the ACHRU-CPP or usual care. At the time of data collection, 53 older adults who had completed the 6-month intervention were approached to participate in the qualitative interviews and 45 accepted (84.9%). The rate of acceptance by site was as follows: Site 1, 100% (10/10); Site 2, 89.9% (8/9); Site 3, 89.9% (8/9); Site 4, 60% (6/10); Site 5, 100% (8/8); and Site 6, 71.4% (5/7). Out of the 45 participants, there was good uptake of home visits with a mean of 3.1 (SD (standard deviation) = 1.5) and group wellness sessions with a mean of 2.7 (SD = 1.9). Where the providers deemed clinically necessary participants received more than the allotted 3

For peer review only - http://bmjopen.bmj.com/site/about/guidelines.xhtml

home visits. Competing commitments such as doctor appointments and lack of interest were barriers to attending group wellness sessions for some participants.

Demographic Characteristics

The mean age of the 45 older adults who participated in interviews was 71.1 years and the mean length of time living with diabetes was 18.8 years (SD = 10.6). Most were female (55.6%), retired from paid work (80%), had Type 2 diabetes (93.3%), and reported 4-6 chronic conditions (44.4%). Hypertension, hyperlipidemia, and osteoarthritis and other arthritis (e.g., rheumatoid arthritis) were the most reported chronic conditions. Table 3 summarizes demographic characteristics of participants.

Table 3. Demographic characteristics of interview participants

**Older Adults (n=45)**	
**Category**	**n (%)**
Age (mean [Standard Deviation]): 71.7 [6.5]	
65-70	26 (57.8)
71-75	7 (15.6)
76+	12 (26.7)
Sex	
Female	25 (55.6)
Male	20 (44.4)
Marital Status	
Married or living with a partner	21 (46.7)
Divorced, never married, separated, or widowed	23 (51.1)
Refused	1 (2.2)
Highest Level of Education	
Completed a graduate or professional degree	6 (13.3)
Completed a bachelor's degree	10 (22.2)
Had some university education or completed a community college, technical college, or postsecondary program	12 (26.7)
Completed secondary school	10 (22.2)
Did not complete secondary school	7 (15.6)
Current Employment Status	
Retired from paid work	36 (80.0)

For peer review only - http://bmjopen.bmj.com/site/about/guidelines.xhtml

Older Adults (n=45)	
**Category**	**n (%)**
Employed full-time	4 (8.9)
Employed part-time	2 (4.4)
Unemployed and looking for work	1 (2.2)
Refused	2 (4.4)
Annual Household Income	
$150,000 or more	2 (4.4)
$100,000 or more, but less than $150,000	2 (4.4)
$50,000 or more, but less than $100,000	12 (26.7)
$20,000 or more, but less than $50,000	16 (35.6)
Less than $20,000	11 (24.4)
Refused	2 (4.4)
Born in Canada	
Yes	31 (68.9)
Ethnic/Racial Group	
White/Caucasian	32 (71.1)
Black	3 (6.7)
Caribbean/Guyanese	3 (6.7)
Filipino	2 (4.4)
First Nations	1 (2.2)
South Asian	1 (2.2)
Southeast Asian	1 (2.2)
Chinese	1 (2.2)
Japanese	1 (2.2)
Language(s) Spoken	
English	37 (82.2)
French	15 (33.3)
Living with Others (e.g., spouse, children, other relative, friend, group setting)	
Yes	27 (60.0)
Type of Diabetes	
Type 1 diabetes	1 (2.2)
Type 2 diabetes	42 (93.3)
Unknown	2 (4.4)
Number of Chronic Conditions (mean [Standard Deviation]): 5.6 [2.9]	
1-3	11 (24.4)
4-6	20 (44.4)
7-9	8 (17.8)
10 +	6 (13.3)
Commonly Reported Chronic Conditions	
Hypertension	34 (75.6)
Hyperlipidemia	27 (60.0)
Osteoarthritis and other arthritis	18 (40.0)
Cardiovascular disease	16 (35.6)
At Least 1 Emergency Room Visit in the Last 6 Months	

For peer review only - http://bmjopen.bmj.com/site/about/guidelines.xhtml

BMJ Open

Older Adults (n=45)	
**Category**	**n (%)**
6 months prior to ACHRU-CPP	8 (17.8)
6-month follow-up	7 (15.6)

Themes

Themes were grouped into two categories, experiences, and perceived impacts of the ACHRU-CPP. Table 4 provides an overview of themes. The words in italics that label the theme are taken verbatim from transcripts. Similarly, participant quotes in the narrative that follows are noted in italics and identified by OA for older adult, # for site number, and ### for participant number.

Table 4. Themes of older adult experiences and perceived impacts with the ACHRU-CPP

**Experiences with the ACHRU-CPP**
• In-depth dialogue with "*professional friends*"<br>• Socialized with *"people with the same type of health problems"*<br>• Person-centred care by *"more than one knowledgeable person"*<br>• Ongoing contact with providers so *"you are not alone"*<br>• Need to address ethnic/cultural differences through a *"personal session"*
**Perceived Impacts of the ACHRU-CPP**
• Improved diabetes self-management behaviours: *"make more proactive steps"*<br>• Added connection to health and social support services *"that could help me"*

For peer review only - http://bmjopen.bmj.com/site/about/guidelines.xhtml

Experiences with the ACHRU-CPP

Overall, older adults reported positive experiences with the ACHRU-CPP. They experienced: (a) in-depth dialogue with "*professional friends*"; (b) socialized with *"people with the same type of health problems"*; (c) person-centred care by *"more than one knowledgeable person";* (d) ongoing contact with providers so *"you are not alone";* and identified the (e) need to address ethnic/cultural differences: *"eating has to do with seasons".*

In-depth dialogue with *"professional friends".* In-person home and virtual visits were perceived by older adults as more relaxed compared to clinic visits and provided opportunities for in-depth dialogue about health and social issues with providers. "*The home visits are more relaxed, if you were at a clinic, you got a time slot you got to meet whatever is transacted in that timeframe"* (OA_1_152). Findings revealed that older adults had a lot of concerns and questions regarding diabetes and other chronic conditions that were often left unanswered by providers due to the nature of clinic visits that are limited by time and ailment. Home visits helped to build trust between older adults and the providers, which facilitated the exploration of concerns and needs beyond diabetes such as safe housing and transportation issues. "*I felt I could trust her [nutritionist]*" (OA_5_037). Providers were approachable and understanding of older adults' situations and were considered as friends and confidants. *"They [providers] were professional friends"* (OA_2_242). Their approach was especially important when discussing sensitive topics such as mental health concerns. "*I had my sick niece calling me and that was stressing me, so she [nurse] said to "Let go. When we are stressed, that's not always good". So how to manage my stress"* (OA_6_023).

Socialized with *"people with the same type of health problems".* Older adults and caregivers perceived that group wellness sessions helped them meet others who understood what

For peer review only - http://bmjopen.bmj.com/site/about/guidelines.xhtml

328 it is like to live with diabetes and other chronic conditions. *"The fact of socializing with other*
329 *people with the same types of health problems as we do"* (OA_5_037). Learning about the
330 burden that others experience with their conditions encouraged older adults to support each other
331 and express their frustrations. The sessions provided opportunities for group exercises which
332 provided peer motivation. The group sessions were particularly helpful for older adults who were
333 socially isolated, and some older adults became friends because of the sessions.

334 **Person-centred care by *"more than one knowledgeable person".*** Older adults
335 appreciated that they received person-centred care from a team of providers through the
336 ACHRU-CPP to discuss diabetes, their other chronic conditions, and social concerns. Some
337 older adults perceived that in usual care providers at times delivered care in silos or independent
338 of other disciplines. Older adults valued providers working collaboratively to meet their needs.
339 *"It was good that they worked in a team. More than one knowledgeable person. That was*
340 *important"* (OA_2_242). They felt that providers were listening to their concerns and that, prior
341 to meeting with the intervention team, it was difficult to find the right person to talk to about
342 diabetes.

343 *I enjoyed having them come to visit. I don't talk to a lot of people about my diabetes because*
344 *I don't feel it's that complicated, but nobody really wants to listen about your health issues.*
345 (OA_3_032)
346
347 Providers supported the management of other conditions in addition to diabetes. *"I was having*
348 *troubles with my bowels, but we got that regulated and it's good"* (OA_4_075).

349 **Ongoing contact with providers so *"you are not alone".*** Providers made older adults
350 feel that someone was concerned about their well-being. "*It's not as if we are just left alone with*
351 *our problems. What you are doing is very good; continue"* (OA_5_128). Managing diabetes and
352 older adults felt burdensome for older adults and they appreciated regular contact with providers.

For peer review only - http://bmjopen.bmj.com/site/about/guidelines.xhtml

For peer review only

Follow-up phone calls were well received by older adults, especially by those living alone or with little support, and ensured that they "*haven't fallen through the cracks*" (OA_3_058). The ongoing follow-up with the team reinforced familiar information that older adults had forgotten to put into practice over time. A few participants reported that once the intervention stopped at the 6-month period they felt that there was a break in the social connection with peers and formal providers.

Need to address ethnic/cultural differences through a *"personal session"*. Some ethnic groups may have language barriers and be *"very shy and they don't approach people unless someone else pushes them to go [join programs]"* (OA_1_061). Some older adults perceived that individual wellness sessions with providers may be helpful for those with language barriers. *"Some of them had a bit of a language problem. I think a personal session would be much more helpful"* (OA_2_086). There is a need to allow dedicated time for older adults to share their cultural practices during interactions with peers and the intervention team.

> *One of the things I learned about myself from my community [Indigenous community] and my family is that eating has to do with seasons...Your year-round diet has to do with what's available to you...I mentioned that one time in the group [Group Wellness Sessions]and they thought that had nothing to do with what the topics were.* (OA_2_013)

Perceived Impacts

Older adults perceived that the ACHRU-CPP had positive impacts on their health and well-being as a result of: (a) improved diabetes self-management behaviours: *"make more proactive steps"* and (b) added connection to health and social support services *"that could help me"*.

Improved diabetes self-management behaviours: *"make more proactive steps"*. Older adults felt that the ACHRU-CPP helped them to take more action in preventing hypoglycemia and hyperglycemia and decreasing their blood pressure. They recognized that reaching a level of

For peer review only - http://bmjopen.bmj.com/site/about/guidelines.xhtml

effective self-management of chronic conditions can be complicated and they appreciated the support provided through the ACHRU-CPP. They reported that providers helped them to be alert to complications that can arise from poor diabetes care.

> *What [the nurse] and [dietitian] caused me to be concerned about is to make more proactive steps, to watch out for those low blood sugars. I really didn't realize how badly they could affect you. Shaking and double vision is one thing but not being able to drive, that's quite another thing.* (OA_2_242)

Older adults indicated that they gained nutritional knowledge by participating in the ACHRU-CPP. "*I improved it [eating habits]. I had to eat more fruit and vegetables...and after that, I had to hydrate myself more and add more fibre to my diet"* (OA_5_027). Older adults perceived that changes made to their diet could lead to multiple benefits including weight loss and decreased sugar levels. "*...drinking more water and diet, I think that's what was important, and I lost weight at the same time. By eating well, fewer treats, being more careful, the sugar levels were lower"* (OA_6_005).

Older adults perceived they were able to build more muscle mass and lose weight and experienced less difficulty in climbing stairs. Some older adults felt that they were not exercising enough prior to participating in the ACHRU-CPP and perceived that the providers helped them to meet their activity goals.

> *They really helped me with the exercise piece. I had poor balance. It was the [nurse and dietitian] that really said 'why don't you try doing this? I'll give you a call this week and see if you got out to do your walk', and then I'd promise them that I would start journaling my steps so little by little I started increasing my exercise.* (OA_2_247)

Added connection to health and social support services *"that could help me".* Older adults indicated that they were referred to and connected with health and social support services (e.g., food bank, exercise program, smoking cessation, home care, social work, arts program). Living well with diabetes and other chronic conditions were perceived by older adults as

For peer review only - http://bmjopen.bmj.com/site/about/guidelines.xhtml

BMJ Open

requiring more than just medical care. Some older adults required supports to meet their basic needs, as they were not able to afford groceries or travel far distances for groceries and medications. "*She [nurse] referred me [for medication delivery]*" (OA_1_061). Some older adults required mental health support to enhance their ability to manage diabetes and other chronic conditions. The intervention team followed-up with older adults after making referrals to ensure that they were connected. Older adults were referred to local community resources that offered free or low-cost services. "*They told me I could go to [name of community centre] to do exercise"* (OA_4_016). Prior to participating in the ACHRU-CPP older adults indicated that they had seldom been referred to programs outside of the clinic and therefore they were not aware of available community resources.

Older adults found it important to be aware of publicly-funded resources, such as tax rebates and housing options, in case they or their loved one required these in the future. By attending group wellness sessions, they learned about the types of programs the community partner sites had to offer. *"They [community partner site] have virtual classes and they're all free. They have special classes just for seniors"* (OA_2_013). Despite the mostly positive impacts of being connected with relevant health and social services, some participants reported that not all of their needs were met at the group wellness sessions. For example, some did not qualify for certain financial assistance programs. *"They just said you have to qualify [for financial assistance] because...you have to apply for this, you have to apply for this"* (OA_1_058). Some participants reported that it was difficult to absorb all of the information presented and not all of the information was relevant to their situation. It was challenging to tailor group wellness sessions to individual needs compared to home visits which allowed further individualized tailoring.

For peer review only - http://bmjopen.bmj.com/site/about/guidelines.xhtml

Discussion

Key findings of this study were that the ACHRU-CPP increased in-depth dialogue with '*professional friends'* and provided person-centred care and ongoing contact with providers to prevent feelings of being alone. Group interactions brought together participants with the same type of health issues and provided peer motivation. Participants identified that the program would benefit from adaptations to address cultural and language differences among older adults living with diabetes and other conditions in Canada.

Older adults with multimorbidity and diabetes face significant burden in managing their chronic conditions, which challenge their self-care and adversely affect their overall quality of life [36]. In addition to managing diabetes symptoms and complications (e.g., hypoglycemia and hyperglycemia, visual impairment, neuropathy) which negatively impact their physical functioning, older adults with diabetes often experience psychological burden associated with complex medication and diet regimens (e.g., medication and insulin management and glycemic control) [36]. Added pressures include financial burden, food insecurity, social isolation, lack of social support, as well as frailty, and the burden of comorbid chronic conditions, e.g., anxiety, depression [36]. Several of these challenges were experienced by our study participants. The largely positive response to the program could be attributed to the support that participants received in managing the burden associated with living with diabetes and other chronic conditions. High quality care for this population to prevent diabetes distress (i.e., challenges faced when dealing with the demands of diabetes), requires good communication and trusting relationships with providers, social and peer support, and self-management education [37]. A novel finding of this study was that the ACHRU-CPP was perceived by older adults from three

For peer review only - http://bmjopen.bmj.com/site/about/guidelines.xhtml

Canadian provinces to positively impact their self-management practices of diabetes and MCC by helping to address their broad health and social needs. This has not been documented before in similar studies [19, 20]. This may be because the ACHRU-CPP was longer and more person-centred compared to other interventions. Interventionists were able to directly assess the home context and understand the impacts of social determinants of health. Mental health concerns and lack of support can impact the ability of older adults to effectively manage diabetes and lead to severe hypoglycemia, elevated HbA1c levels, a greater number of missed insulin doses, and a higher risk for diabetic ketoacidosis and mortality [38, 39, 3].

When healthcare providers recommend lifestyle changes, they need to recognize that social determinants of health such as housing, food security, social relationships, and financial stability have an impact on older adults' abilities to enact them [40]. In the current study, the intervention team assessed the social determinants of health and found ways to address them, such as by linking older adults with relevant community resources, to help overcome barriers to self-management. The intervention team targeted health literacy of older adults and caregivers through education, capacity building, and opportunities for dialogue among peers and experts. Community-based interventions were found to be most effective for Type 2 diabetes self-management compared to other interventions [21].

In the current study, older adults appreciated receiving person-centred care supported by a team of providers from primary care and community sectors and the engagement of providers outside of the intervention team (such as social workers). Due to the complex nature of diabetes and MCC, interprofessional collaboration has been found to lead to positive outcomes for persons with Type 2 diabetes, such as improvements in HbA1c levels, regular testing of blood glucose levels, and smoking cessation [41, 42].

For peer review only - http://bmjopen.bmj.com/site/about/guidelines.xhtml

BMJ Open

What is unique about this study is the partnership between healthcare providers and a Program Coordinator from a local community partner site. These health and social services can be underutilized if healthcare providers are not aware of them. As per the Quintuple Aim [23] there is a need to optimize the use of existing community-based services for patients, address any barriers to accessing these services, and for strong coordination of services [43].

Person-centred care was perceived to be key strength of the ACHRU-CPP that enabled older adults to improve self-management practices related to diabetes and MCC. In working towards a person-centred learning health system, defined as a health system that integrates internal data, patient experience, and research evidence [44], the priorities and experiences of older adults should be regularly reported in data systems so that services that meet their needs are developed and evaluated as part of continuous quality improvement processes [38]. As seen in this study, patient experience can be improved by having a provider connect patients with other interdisciplinary health and social care providers to ensure that smooth transitions between services occur [45].

In practice and policy, there is a need for integrated care delivery models that leverage community partnerships to help fill gaps in meeting the complex health and social needs of older adults with diabetes. To advance Quintuple Aim outcomes [23], it is critical to assess patient experiences with receiving healthcare services as part of intervention research and practice to improve health system performance. The strengths of the study include its rigorous qualitative design and large sample size, and the inclusion of diverse participants with regards to sex, marital status, and annual income from multiple sites across Canada. A limitation of the study was related to the sample as there was a lack of cultural diversity and under-representation of older adults from marginalized communities. A Diabetes Canada roundtable of key stakeholders

For peer review only - http://bmjopen.bmj.com/site/about/guidelines.xhtml

recently emphasized the need to implement community-based interventions [46], such as the ACHRU-CPP, to better support older adults with diabetes and MCC living in marginalized communities. The need for community-based interventions is based on the premise that racial and socioeconomic disparities disproportionally affect them and put them at an increased risk for diabetes complications and mortality [47].

Conclusion

Overall, older adults with diabetes and MCC reported a positive experience and felt that the ACHRU-CPP had a positive impact in supporting diabetes self-management. Study findings reveal the need to ensure that older adults receive ongoing support and contact with a collaborative team of primary care and community providers to better meet the complex needs associated with daily self-management of diabetes and MCC. Results also shine light on the broader social context that constitutes the life world of older adults and how chronic disease self-management interventions need to address these contexts comprehensively through tailoring to individual circumstances. It is our hope that these findings will help usher in a new era of contextually informed person-centred care.

Acknowledgements

We thank the older adults and caregivers who participated in this study, as well as the nurses, dietitians, nutritionists and community program coordinators who provided the intervention. We also thank the managers of intervention teams, the recruiters, research assistants, and the study sites for their support of this study. Thanks to Robyn Connors for conducting interviews with study participants. We thank the research team in the Aging,

For peer review only - http://bmjopen.bmj.com/site/about/guidelines.xhtml

Community and Health Research Unit (https://achru.mcmaster.ca/) for supporting this study. We would also like to thank the Community Advisory Board members, including people with lived experience with diabetes, who supported local implementation of the project in each province.

We acknowledge the following authors who are members of the **ACHRU-CPP Research Team**: **Jenny Ploeg**, School of Nursing, Faculty of Health Sciences, McMaster University, Hamilton, Ontario, Canada; **Maureen Markle-Reid**, School of Nursing, Faculty of Health Sciences, McMaster University, Hamilton, Ontario, Canada; **Ruta Valaitis**, School of Nursing, Faculty of Health Sciences, McMaster University, Hamilton, Ontario, Canada; **Kathryn Fisher,** School of Nursing, Aging, Community and Health Research Unit, Faculty of Health Sciences, McMaster University, Hamilton, Ontario, Canada; **Rebecca Ganann**, School of Nursing, Aging, Community and Health Research Unit, Faculty of Health Sciences, McMaster University, Hamilton, Ontario, Canada; **Johanne Blais**, Department of Family Medicine and Emergency Medicine, Faculty of Medicine, Université Laval, Quebec City, Quebec; **Andrea Gruneir**, Department of Family Medicine Research Program, University of Alberta, Edmonton, Alberta, Canada; **France Légaré**, VITAM-Centre de recherche en santé durable, Université Laval, Quebec City, Quebec, Canada; **Janet MacIntyre**, Faculty of Nursing, University of Prince Edward Island, Charlottetown, Prince Edward Island, Canada; **William Montelpare**, Department of Applied Human Sciences, Faculty of Science, University of Prince Edward Island, Prince Edward Island, Canada; **Jean-Sébastien Paquette**, Department of Family Medicine and Emergency Medicine, Faculty of Medicine Université Laval, Québec, Canada; **Marie-Eve Poitras**, Department of Family Medicine and Emergency Medicine, Faculty of Medicine and Health Sciences, Université de Sherbrooke Chicoutimi, Quebec, Canada; **Angela Riveroll**, Department of Applied Human Sciences, Faculty of Science, University of Prince

For peer review only

For peer review only - http://bmjopen.bmj.com/site/about/guidelines.xhtml

BMJ Open

Edward Island, Charlottetown, Prince Edward Island, Canada, **Ali Ben Charif,** CubecXpert, Quebec City, Quebec, Canada; **Dean Eurich**, School of Public Health, University of Alberta, Edmonton, Alberta, Canada; **Amiram Gafni**, Department of Health Research Methods, Evidence and Impact, Faculty of Health Sciences, McMaster University, Hamilton, Ontario, Canada; **Gary Lewis**, Department of Medicine and Department of Physiology, University of Toronto, Toronto, Ontario, Canada; **Lynne Mansell**, Patient/Public Research Partner, Alberta, Canada; **Melissa Northwood**, School of Nursing, Aging, Community and Health Research Unit, Faculty of Health Sciences, McMaster University, Hamilton, Ontario, Canada; **Janet Pritchard**, Interdisciplinary Science and Kinesiology, Faculty of Science, McMaster University, Hamilton, Ontario, Canada; **Cheryl Sadowski**, Faculty of Pharmacy and Pharmaceutical Sciences, University of Alberta, Edmonton, Alberta, Canada; **Diana Sherifali**, School of Nursing, Faculty of Health Sciences, McMaster University, Hamilton, Ontario, Canada; **Frank Tang**, Patient/Public Research Partner, Ontario, Canada**; Lehana Thabane,** Department of Health Research Methods, Evidence and Impact, Faculty of Health Sciences, McMaster University, Hamilton, Ontario, Canada; **Ross Upshur**, Bridgepoint Active Healthcare, Toronto, Ontario, Canada; **Tyler Williamson**, Centre for Health Informatics, Cumming School of Medicine and Department of Community Health Sciences, University of Calgary, Calgary, Alberta, Canada; **Marie-Lee Yous,** School of Nursing, Faculty of Health Sciences, McMaster University, Hamilton, Ontario, Canada.

Author Contributions

Conceptualization: JP, MMR, RV, KF, RG, FL, WM.

Formal Analysis: MY, RG, TC, JP.

For peer review only - http://bmjopen.bmj.com/site/about/guidelines.xhtml

Funding Acquisition: JP, MMR, RV, KF, RG, AG, FL, JM, WM.

Investigation: JP, MMR, RV, KF, RG, TC, FL, WM, MY.

Methodology: JP, MMR, RV, KF, RG, MY, FL, WM.

Project Administration: JP, MMR, RV, TC, FL, WM.

Resources: JP, MMR, RV, FL, WM.

Supervision: JP, MMR, RV, RG, TC, FL, WM.

Validation: MY, JP, MMR, RV, KF, RG, TC, RC.

Writing – Original Draft: MY.

Writing – Review and Editing: MY, RG, TC, JP, MMR, RV, KF, FL, JM, WM. The authors read and approved the final manuscript.

Funding

This study is supported, in part, by funding from the Canadian Institutes of Health Research Strategy for Patient-Oriented Research (SPOR) Primary and Integrated Health Care Innovations Network: Programmatic Grants (Funding Reference Number: KPG-156883) in partnership with: Diabetes Action Canada, a Canadian Institutes for Health Research (CIHR) Strategy for Patient-Oriented Research Network in Chronic Disease (project reference #1.1.1ACHR); McMaster Institute for Research on Aging (Hamilton, ON); McMaster University School of Nursing; Réseau-1 Québec; Fonds de Recherche du Québec (FRQS); Scarborough Health Network Foundation. This research was also undertaken, in part, thanks to the funding from Dr. Markle-Reid's Tier 2 CIHR Canada Research Chair. The study was investigator-initiated. The funders of this study had no role in study design, data collection, data analysis, data interpretation or writing the manuscript.

For peer review only - http://bmjopen.bmj.com/site/about/guidelines.xhtml

Competing Interests

None declared.

Availability of Data and Materials

The data for this research consists of questionnaires and interviews. Raw data such as audio-files and interview transcripts cannot be publicly released due to the risk of compromising participant confidentiality related to identification of voices and publicly exposing personal information.

Ethics Statement

Institutional ethics approval was obtained from the following: the Hamilton Integrated Research Ethics Board (#5101); the Scarborough Health Network Research Ethics Board (#NEP-18-014); the Unity Health Toronto Research Ethics Board (#18-336); University of Prince Edward Island Research Ethics Board (#6008019); Prince Edward Island Research Ethics Board (#6008019); and Centre intégré universitaire de santé et de services sociaux (CIUSSS) de la Capitale-Nationale (MP-13-2019-1670). Trained research assistants obtained informed consent from all older adult participants prior to their participation in the study, and all participants received a copy of their consent form, in person or by mail. All participants gave permission to audio-record their interview. All participant information was kept confidential in a secure location (e.g., locked cabinet in a secured office, password-protected encrypted electronic folders), and data were de-identified using unique study IDs. Participants who completed a qualitative interview received a $25.00 gift card as an honorarium.

For peer review only - http://bmjopen.bmj.com/site/about/guidelines.xhtml

References

[1] World Health Organization. Diabetes. Available from: https://www.who.int/en/news-room/fact-sheets/detail/diabetes. 2022. Accessed 3 Jan 2022.
[2] Kirkman MS, Briscoe VJ, Clark N, et al. Diabetes in older adults: A consensus report. *J Am Geriatr Soc* 2012;35(12):2650-64.
[3] Dhaliwal R, Weinstock RS. Management of type 1 diabetes in older adults. *Diabetes Spectr*. 2014;27(1):9-20.
[4] Sinnige J, Braspenning J, Schellevis F, et al. The prevalence of disease clusters in older adults with multiple chronic diseases–a systematic literature review. *PloS one* 2013;8(11):e79641.
[5] Lin PJ, Kent DM, Winn A, et al. Multiple chronic conditions in type 2 diabetes mellitus: prevalence and consequences. *Am J Manag Care* 2015;21(1):e23-34.
[6] Fisher K, Griffith L, Gruneir A, et al. Comorbidity and its relationship with health service use and cost in community-living older adults with diabetes: A population-based study in Ontario, Canada. *Diabetes Res Clin Pract* 2016;122:113-123.
[7] Willi C, Bodenmann P, Ghali WA, et al. Active smoking and the risk of type 2 diabetes: A systematic review and meta-analysis. *JAMA* 2007; 298:2654-64.
[8] Ploeg J, Matthew-Maich N, Fraser K, et al. Managing multiple chronic conditions in the community: A Canadian qualitative study of the experiences of older adults, family caregivers and healthcare providers. *BMC Geriatr* 2017;17.
[9] The Change Foundation. Family caregiver assessment in health care settings, summary of the change foundation's literature review and environment scan project. Toronto, August 2016.
[10] McGilton KS, Vellani S, Yeung L, et al. Identifying and understanding the health and social care needs of older adults with multiple chronic conditions and their caregivers: A scoping review. BMC Geriatr 2018;18(1):1-33.
[11Craig P, Dieppe P, Macintyre S, et al. Developing and evaluating complex interventions: the new Medical Research Council guidance. *BMJ*. 2008;337:1-6.
[12] Whittemore R. A systematic review of the translational research on the diabetes prevention program. *Transl Behav Med* 2011;1:480-91.
[13] Tuomilehto J, Schwarz P, Lindström J. Long-term benefits from lifestyle interventions for type 2 diabetes prevention: Time to expand the efforts. *Diabetes Care* 2011;34:S210-14.
[14] Smith SM, Wallace E, O'Dowd T, et al. Interventions for improving outcomes in patients with multimorbidity in primary care and community settings. *Cochrane Database Syst Rev* 2016;3.
[15] Yoon U, Kwok LL, Magkidis A. Efficacy of lifestyle interventions in reducing diabetes incidence in patients with impaired glucose tolerance: A systematic review of randomized controlled trials. *Metabolism* 2013;62:303-14.
[16] Busetto L, Luijkx KG, Elissen AMJ, et al. Context, mechanisms and outcomes of integrated care for diabetes mellitus type 2: A systematic review. *BMC Health Serv Res* 2016;16:1-14.
[17] Diabetes Prevention Program Research Group. 10-year follow-up of diabetes incidence and weight loss in the diabetes prevention program outcomes study. *Lancet* 2009;374:1677-86.

For peer review only - http://bmjopen.bmj.com/site/about/guidelines.xhtml

[18] Wong KC, Wong FKY, Yeung W, et al. The effect of complex interventions on supporting self-care among community-dwelling older adults: A systematic review and meta-analysis. *Age Ageing* 2017;1-9.
[19] Azami G, Soh KL, Sazlina SG, et al. Effect of a nurse-led diabetes self-management education program on glycosylated hemoglobin among adults with type 2 diabetes. *J Diabetes Res* 2018:1-13.
[20] Chow SK, Wong FK. A randomized controlled trial of a nurse-led case management programme for hospital-discharged older adults with co-morbidities. *J Adv Nurs* 2014;70(10):2257-71.
[21] Dahal PK, Hosseinzadeh H. Association of health literacy and diabetes self-management: A systematic review. *Aust J Prim Health* 2020;25(6):526-33.
[22] Ferris R, Blaum C, Kiwak E, et al. Perspectives of patients, clinicians, and health system leaders on changes needed to improve the health care and outcomes of older adults with multiple chronic conditions. *J Aging Health* 2018;30(5):778-99.
[23] Nundy S, Cooper LA, Mate KS. The Quintuple Aim for health care improvement: A new imperative to advance health equity. *JAMA* 2022;327(6):521–522.
[24] Markle-Reid M, Ploeg J, Fisher K, et al. The Aging, Community and Health Research Unit—Community Partnership Program for older adults with type 2 diabetes and multiple chronic conditions: A feasibility study. *Pilot Feasibility Stud* 2016 2(1):24.
[25] Markle-Reid M, Ploeg J, Fraser KD, et al. Community program improves quality of life and self-management in older adults with diabetes mellitus and comorbidity. J Am Geriatr Soc. 2018 Feb;66(2):263-73.
[26] Miklavcic JJ, Fraser KD, Ploeg J, Markle-Reid M, Fisher K, Gafni A, Griffith LE, Hirst S, Sadowski CA, Thabane L, Triscott JA. Effectiveness of a community program for older adults with type 2 diabetes and multimorbidity: A pragmatic randomized controlled trial. BMC Geriatr. 2020 Dec;20(1):1-14.
[27] Fisher K, Markle-Reid M, Ploeg J, Bartholomew A, Griffith LE, Gafni A, Thabane L, Yous ML. Self-management program versus usual care for community-dwelling older adults with multimorbidity: A pragmatic randomized controlled trial in Ontario, Canada. J Comorb. 2020 Oct 14;20(174):1-14.
[28] Ploeg J, Markle-Reid M, Valaitis R, et al. The Aging, Community and Health Research Unit Community Partnership Program for older adults with diabetes and multiple chronic conditions: Study protocol for a randomized controlled trial. *BMC Geriatrics* 2022;22(99):1-22.
[29] Sandelowski M. Whatever happened to qualitative description? Res Nurs health. 2000;23(4):334-40.
[30] Sandelowski M. What's in a name? Qualitative description revisited. Res Nurs Health. 2010 Feb;33(1):77-84.
[31] Patton MQ. Qualitative evaluation and research methods. Beverly Hills, CA: SAGE, 1990.
[32] Braun V, Clarke V. Successful qualitative research: A practical guide for beginners. London, England: SAGE, 2013.
[33] Damschroder LJ, Aron DC, Keith RE, et al. Fostering implementation of health services research findings into practice: A consolidated framework for advancing implementation science. Implement Sci. 2009 Dec;4(1):1-5.
[34] QSR International Pty Ltd. NVivo (Version 12). 2018. Available from: https://www.qsrinternational.com/nvivo-qualitative-data-analysis-software/home
[35] Lincoln YS, Guba EG. Naturalistic inquiry. Sage Publications, 1985.

For peer review only - http://bmjopen.bmj.com/site/about/guidelines.xhtml

BMJ Open

[36] Sayyed Kassem L, Aron DC. The assessment and management of quality of life of older adults with diabetes mellitus. Expert Rev Endocrinol Metab. 2020 Mar 3;15(2):71-81.
[37] Skinner TC, Joensen L, Parkin T. Twenty-five years of diabetes distress research. Diab Med. 2020 Mar;37(3):393-400.
[38] Robinson DJ, Coons M, Haensel H, et al. Diabetes and mental health. *Can J Diabetes* 2018 Apr 1;42:S130-41.
[39] Lynch CP, Gebregziabher M, Zhao Y, et al. Impact of medical and psychiatric multi-morbidity on mortality in diabetes: emerging evidence. *BMC Endocr Disord* 2014;14(1):1-8.
[40] Kuluski K, Guilcher SJ. Toward a person-centred learning health system: Understanding value from the perspectives of patients and caregivers. *Healthc Pap* 2019;18(4):36-46.
[41] Hellquist K, Bradley R, Grambart S, et al. Collaborative practice benefits patients: An examination of interprofessional approaches to diabetes care. *Health Interprofessional Pract* 2012;1(eP1017).
[42] O'Connor PJ, Desai J, Solberg LI, et al. Randomized trial of quality improvement intervention to improve diabetes care in primary care settings. *Diabetes Care* 2005;28,1890–97.
[43] Valaitis RK, Wong ST, MacDonald M, et al. Addressing quadruple aims through primary care and public health collaboration: ten Canadian case studies. BMC Public Health 2020;20(1):1-6.
[44] Agency for Healthcare Research and Quality. About learning health systems. 2019. Available from: https://www.ahrq.gov/learning-health-systems/about.html. Accessed 12 Feb 2022.
[45] Rapid Improvement Support and Exchange. RISE brief 1. OHT (Ontario Health Team) building blocks. 2019. Available from: https://www.mcmasterforum.org/docs/default-source/rise-docs/rise-briefs/rb1_oht-building-blocks.pdf?sfvrsn=71b154d5_27. Accessed 12 Feb 2022.
[46] Diabetes Canada. Summary of Diabetes Canada Diabetes 360˚ Ontario roundtable. 2019. Available from: https://www.diabetes.ca/DiabetesCanadaWebsite/media/Advocacy-and-Policy/Advocacy%20Reports/Ontario-Diabetes-360-Strategy_Roundtable-Summary_FINAL.pdf. Accessed 12 Feb 2022.
[47] Clements JM, West BT, Yaker Z, et al. Disparities in diabetes-related multiple chronic conditions and mortality: The influence of race. *Diabetes Res Clin Pract* 2020;159(107984):1-19.

For peer review only - http://bmjopen.bmj.com/site/about/guidelines.xhtml

COREQ (COnsolidated criteria for REporting Qualitative research) Checklist

A checklist of items that should be included in reports of qualitative research. You must report the page number in your manuscript where you consider each of the items listed in this checklist. If you have not included this information, either revise your manuscript accordingly before submitting or note N/A.

Topic	Item No.	Guide Questions/Description	Reported on Page No.
**Domain 1: Research team and reflexivity**			
Personal characteristics			
Interviewer/facilitator	1	Which author/s conducted the interview or focus group?	
Credentials	2	What were the researcher's credentials? E.g. PhD, MD	
Occupation	3	What was their occupation at the time of the study?	
Gender	4	Was the researcher male or female?	
Experience and training	5	What experience or training did the researcher have?	
Relationship with participants			
Relationship established	6	Was a relationship established prior to study commencement?	
Participant knowledge of the interviewer	7	What did the participants know about the researcher? e.g. personal goals, reasons for doing the research	
Interviewer characteristics	8	What characteristics were reported about the inter viewer/facilitator? e.g. Bias, assumptions, reasons and interests in the research topic	
**Domain 2: Study design**			
Theoretical framework			
Methodological orientation and Theory	9	What methodological orientation was stated to underpin the study? e.g. grounded theory, discourse analysis, ethnography, phenomenology, content analysis	
Participant selection			
Sampling	10	How were participants selected? e.g. purposive, convenience, consecutive, snowball	
Method of approach	11	How were participants approached? e.g. face-to-face, telephone, mail, email	
Sample size	12	How many participants were in the study?	
Non-participation	13	How many people refused to participate or dropped out? Reasons?	
Setting			
Setting of data collection	14	Where was the data collected? e.g. home, clinic, workplace	
Presence of non-participants	15	Was anyone else present besides the participants and researchers?	
Description of sample	16	What are the important characteristics of the sample? e.g. demographic data, date	
Data collection			
Interview guide	17	Were questions, prompts, guides provided by the authors? Was it pilot tested?	
Repeat interviews	18	Were repeat inter views carried out? If yes, how many?	
Audio/visual recording	19	Did the research use audio or visual recording to collect the data?	
Field notes	20	Were field notes made during and/or after the inter view or focus group?	
Duration	21	What was the duration of the inter views or focus group?	
Data saturation	22	Was data saturation discussed?	
Transcripts returned	23	Were transcripts returned to participants for comment and/or	

For peer review only

Topic	Item No.	Guide Questions/Description	Reported on Page No.
		correction?	
**Domain 3: analysis and findings**			
Data analysis			
Number of data coders	24	How many data coders coded the data?	
Description of the coding tree	25	Did authors provide a description of the coding tree?	
Derivation of themes	26	Were themes identified in advance or derived from the data?	
Software	27	What software, if applicable, was used to manage the data?	
Participant checking	28	Did participants provide feedback on the findings?	
Reporting			
Quotations presented	29	Were participant quotations presented to illustrate the themes/findings? Was each quotation identified? e.g. participant number	
Data and findings consistent	30	Was there consistency between the data presented and the findings?	
Clarity of major themes	31	Were major themes clearly presented in the findings?	
Clarity of minor themes	32	Is there a description of diverse cases or discussion of minor themes?	

Developed from: Tong A, Sainsbury P, Craig J. Consolidated criteria for reporting qualitative research (COREQ): a 32-item checklist for interviews and focus groups. *International Journal for Quality in Health Care*. 2007. Volume 19, Number 6: pp. 349 – 357

Once you have completed this checklist, please save a copy and upload it as part of your submission. DO NOT include this checklist as part of the main manuscript document. It must be uploaded as a separate file.

For peer review only

BMJ Open

Older Adults' Experiences and Perceived Impacts of the Aging, Community and Health Research Unit – Community Partnership Program (ACHRU-CPP) for Diabetes Self-Management in Canada: A Qualitative Descriptive Study

Journal:	*BMJ Open*
Manuscript ID	bmjopen-2022-068694.R2
Article Type:	Original research
Date Submitted by the Author:	06-Mar-2023
Complete List of Authors:	Yous, Marie-Lee; McMaster University Faculty of Health Sciences, School of Nursing<br>Ganann, Rebecca; McMaster University Faculty of Health Sciences, School of Nursing, Aging, Community and Health Research Unit<br>Ploeg, Jenny ; McMaster University Faculty of Health Sciences, School of Nursing, Aging, Community and Health Research Unit<br>Markle-Reid, Maureen; McMaster University Faculty of Health Sciences, School of Nursing, Aging, Community and Health Research Unit<br>Northwood, Melissa ; McMaster University Faculty of Health Sciences, School of Nursing, Aging, Community and Health Research Unit<br>Fisher, Kathryn ; McMaster University Faculty of Health Sciences, School of Nursing, Aging, Community and Health Research Unit<br>Valaitis, Ruta; McMaster University Faculty of Health Sciences, School of Nursing, Aging, Community and Health Research Unit<br>Chambers, Tracey; McMaster University Faculty of Health Sciences, School of Nursing, Aging, Community and Health Research Unit<br>Montelpare, William; University of Prince Edward Island, Department of Applied Human Sciences, Faculty of Science<br>Légaré, France; VITAM Centre de recherche en santé durable; Laval University, Department of Family and Emergency Medicine, Faculty of Medicine<br>Beleno, Ron; McMaster University Faculty of Health Sciences, School of Nursing, Aging, Community and Health Research Unit<br>Gaudet, Gary; University of Prince Edward Island<br>Giacometti, Luisa; McMaster University Faculty of Health Sciences, School of Nursing, Aging, Community and Health Research Unit<br>Levely, Deborah; McMaster University Faculty of Health Sciences, School of Nursing, Aging, Community and Health Research Unit<br>Lindsay, Craig; McMaster University Faculty of Health Sciences, School of Nursing, Aging, Community and Health Research Unit<br>Morrison, Allan; University of Prince Edward Island<br>Tang, Frank; McMaster University Faculty of Health Sciences, School of Nursing, Aging, Community and Health Research Unit<br>ACHRU-CPP Research Team, on behalf of the; McMaster University Faculty of Health Sciences, School of Nursing
**Primary Subject Heading**:	Diabetes and endocrinology

Secondary Subject Heading:	Qualitative research
Keywords:	QUALITATIVE RESEARCH, PRIMARY CARE, DIABETES & ENDOCRINOLOGY

SCHOLARONE™ Manuscripts

I, the Submitting Author has the right to grant and does grant on behalf of all authors of the Work (as defined in the below author licence), an exclusive licence and/or a non-exclusive licence for contributions from authors who are: i) UK Crown employees; ii) where BMJ has agreed a CC-BY licence shall apply, and/or iii) in accordance with the terms applicable for US Federal Government officers or employees acting as part of their official duties; on a worldwide, perpetual, irrevocable, royalty-free basis to BMJ Publishing Group Ltd ("BMJ") its licensees and where the relevant Journal is co-owned by BMJ to the co-owners of the Journal, to publish the Work in this journal and any other BMJ products and to exploit all rights, as set out in our licence.

The Submitting Author accepts and understands that any supply made under these terms is made by BMJ to the Submitting Author unless you are acting as an employee on behalf of your employer or a postgraduate student of an affiliated institution which is paying any applicable article publishing charge ("APC") for Open Access articles. Where the Submitting Author wishes to make the Work available on an Open Access basis (and intends to pay the relevant APC), the terms of reuse of such Open Access shall be governed by a Creative Commons licence – details of these licences and which Creative Commons licence will apply to this Work are set out in our licence referred to above.

Other than as permitted in any relevant BMJ Author's Self Archiving Policies, I confirm this Work has not been accepted for publication elsewhere, is not being considered for publication elsewhere and does not duplicate material already published. I confirm all authors consent to publication of this Work and authorise the granting of this licence.

For peer review only

BMJ Open

Original Research

Title: Older Adults' Experiences and Perceived Impacts of the Aging, Community and Health Research Unit – Community Partnership Program (ACHRU-CPP) for Diabetes Self-Management in Canada: A Qualitative Descriptive Study

Corresponding Author: Marie-Lee Yous, School of Nursing, Aging, Community and Health Research Unit, Faculty of Health Sciences, McMaster University, 1280 Main Street West, Room HSC 3N25, Hamilton, Ontario L8S 4K1, Canada, Email: yousm@mcmaster.ca; ORCID: 0000-0002-4271-0401

Marie-Lee Yous, School of Nursing, Aging, Community and Health Research Unit, Faculty of Health Sciences, McMaster University, Hamilton, Ontario, Canada, email: yousm@mcmaster.ca ORCID: 0000-0002-4271-0401

Rebecca Ganann, School of Nursing, Aging, Community and Health Research Unit, Faculty of Health Sciences, McMaster University, email: gananrl@mcmaster.ca ORCID: 0000-0002-7566-8932

Jenny Ploeg, School of Nursing, Aging, Community and Health Research Unit, Faculty of Health Sciences, McMaster University, Hamilton, Ontario, Canada, email: ploegj@mcmaster.ca ORCID: 0000-0001-8168-8449

Maureen Markle-Reid, School of Nursing, Aging, Community and Health Research Unit, Faculty of Health Sciences, McMaster University, Hamilton, Ontario, Canada, email: mreid@mcmaster.ca ORCID: 0000-0002-4019-7077

Melissa Northwood, School of Nursing, Aging, Community and Health Research Unit, Faculty of Health Sciences, McMaster University, Hamilton, Ontario, Canada, email: northwm@mcmaster.ca ORCID: 0000-0001-5043-8068

Kathryn Fisher, School of Nursing, Aging, Community and Health Research Unit, Faculty of Health Sciences, McMaster University, Hamilton, Ontario, Canada fisheka@mcmaster.ca ORCID: 0000-0001-8342-1238

Ruta Valaitis, School of Nursing, Aging, Community and Health Research Unit, Faculty of Health Sciences, McMaster University, Hamilton, Ontario, Canada, email: valaitis@mcmaster.ca ORCID: 0000-0002-3117-0542

Tracey Chambers, School of Nursing, Aging, Community and Health Research Unit, Faculty of Health Sciences, McMaster University, Hamilton, Ontario, Canada, email: chambt@mcmaster.ca ORCID: 0000-0002-9325-6894

William Montelpare, Margaret and Wallace McCain Chair in Human Development and Health, Department of Applied Human Sciences, Faculty of Science, University of Prince Edward

For peer review only - http://bmjopen.bmj.com/site/about/guidelines.xhtml

Island, Charlottetown, Prince Edward Island, Canada, email: wmontelpare@upei.ca ORCID: 0000-0002-4167-4613

France Légaré, VITAM-Centre de recherche en santé durable, Université Laval, Québec City, Québec, Canada, email: France.legare@fmed.ulaval.ca ORCID: 0000-0002-2296-6696

Ron Beleno, Patient/Public Research Partner, Aging, Community and Health Research Unit, Faculty of Health Sciences, McMaster University, Hamilton, Ontario, Canada email: ron@rb33.com

Gary Gaudet, Patient/Public Research Partner, University of Prince Edward Island, Charlottetown, Prince Edward Island, Canada, email: l.ggaudet@bellaliant.net

Luisa Giacometti, Patient/Public Research Partner, School of Nursing, Aging, Community and Health Research Unit, Faculty of Health Sciences, McMaster University, Hamilton, Ontario, Canada, email: luisag@bell.net

Deborah Levely, Patient/Public Research Partner, School of Nursing, Aging, Community and Health Research Unit, Faculty of Health Sciences, McMaster University, Hamilton, Ontario, Canada, email: deborah.levely@rogers.com

Craig Lindsay, Patient/Public Research Partner, School of Nursing, Aging, Community and Health Research Unit, Faculty of Health Sciences, McMaster University, Hamilton, Ontario, Canada, email: lindsay.cr@gmail.com

Allan Morrison, Patient/Public Research Partner, University of Prince Edward Island, Charlottetown, Prince Edward Island, Canada, email: allan.morrison62.am@gmail.com

Frank Tang, Patient/Public Research Partner, School of Nursing, Aging, Community and Health Research Unit, Faculty of Health Sciences, McMaster University, Hamilton, Ontario, Canada, email: frankmtang@gmail.com

On behalf of the ACHRU-CPP Research Team

Word count excluding title page, abstract, references, figures and tables **(max 4,000**): 4,721

For peer review only - http://bmjopen.bmj.com/site/about/guidelines.xhtml

BMJ Open

Abstract (max 300 words): 300 words

Objectives: To assess the experiences and perceived impacts of the Aging, Community and Health Research Unit – Community Partnership Program (ACHRU-CPP) from the perspectives of older adults with diabetes and other chronic conditions. The ACHRU-CPP is a complex 6-month self-management evidence-based intervention for community-living older adults aged 65 years or older with Type 1 or Type 2 diabetes and at least one other chronic condition. It includes home and phone visits, care coordination, system navigation support, caregiver support, and group wellness sessions delivered by a nurse, dietitian or nutritionist, and community program coordinator.

Design: Qualitative descriptive design embedded within a randomized controlled trial was used.

Setting: Six trial sites offering primary care services from three Canadian provinces (i.e., Ontario, Quebec, and Prince Edward Island) were included.

Participants: The sample was 45 community-living older adults aged 65 years or older with diabetes and at least one other chronic condition.

Methods: Participants completed semi-structured post-intervention interviews by phone in English or French. The analytical process followed Braun and Clarke's experiential thematic analysis framework. Patient partners informed study design and interpretation.

Results: The mean age of older adults was 71.7 years, and the mean length of time living with diabetes was 18.8 years. Older adults reported positive experiences with the ACHRU-CPP that supported diabetes self-management such as, improved knowledge in managing diabetes and other chronic conditions, enhanced physical activity and function, improved eating habits, and opportunities for socialization. They reported being connected to community resources by the intervention team to address social determinants of health and support self-management.

For peer review only - http://bmjopen.bmj.com/site/about/guidelines.xhtml

BMJ Open

Conclusions: Older adults perceived that a 6-month person-centred intervention collaboratively delivered by a team of health and social care providers helped support chronic disease self-management. There is a need for providers to help older adults connect with available health and social services in the community.

Strengths and limitations of this study

- This study included a rigorous qualitative design with a large sample size.
- A rigorous analytic method was used involving multiple researchers with expertise in primary care, qualitative, ageing, and diabetes research, as well as programme evaluation.
- Patient and public research partners were involved in designing the intervention, informing the study design and interview guides, interpreting the results, and developing the manuscript.
- A limitation of the study was related to the sample as there was a lack of cultural diversity with regards to ethnicity and under-representation of older adults from marginalized communities.

Keywords: diabetes self-management, older adults, community, primary care, qualitative

For peer review only

For peer review only - http://bmjopen.bmj.com/site/about/guidelines.xhtml

Introduction

As of 2022, approximately 422 million people have been diagnosed with diabetes mellitus worldwide [1]. Older adults are more likely to have Type 2 diabetes than younger adults [2] and are at risk for hypoglycemia which can adversely affect cognition, vision, hearing, mobility, and mental health [3] as well as self-care activities including exercise and diet. More than 40% of older adults with diabetes have three or more chronic conditions [4], including hyperlipidemia, hypertension, asthma, chronic obstructive pulmonary disease, chronic kidney disease, arthritis, and heart failure [5]. Following management plans for one condition may be challenging due to symptoms or conflicting guidelines from another condition. Higher burden associated with the presence of multiple chronic conditions (MCC) has been linked to higher risk for mortality, decreased physical and mental functioning, and increased health services use [2, 6, 7]. Community-dwelling older adults with MCC are highly reliant on family/friend caregivers for support [8], which can lead to poor mental and physical health, and financial losses among caregivers [9]. Caregivers' unmet needs [10] can lead to their increased use of hospital and emergency services.

Complex health interventions are defined as having multiple interacting components [11]. For older adults with diabetes, complex health interventions such as peer support programs, have demonstrated positive effects in managing their complex needs, sustaining lifestyle changes, and achieving health benefits [12-17]. Complex interventions that target self-care and incorporate opportunities for peer-to-peer discussions among community-dwelling older adults can improve their mental and physical health and reduce falls [18, 19]. Nurse-led self-management programs for diabetes and other chronic conditions can lead to improvements in self-rated health, glycated hemoglobin (HbA1c) values, blood pressure, weight, and self-management behaviours [19, 20].

For peer review only - http://bmjopen.bmj.com/site/about/guidelines.xhtml

BMJ Open

Providing diabetes self-management programs through partnerships between primary care and community organizations (e.g., senior centres, YMCA) supports program uptake, implementation, and sustainability [12], which can lead to improved health literacy (i.e., being able to locate, read and understand health information for informed decision-making). In a systematic review of randomized controlled trials (RCTs), health literacy was instrumental in enhancing diabetes knowledge, self-efficacy, and physical activity [21].

Receiving care from multiple providers from different health and community settings can lead to fragmented care for older adults, as referrals across organizations are often not well integrated [22]. Seamless care coordination and system navigation for older adults with MCC remain high priorities for this population. There is a need for innovative programs for older adults that focus on the Quintuple Aims of high-quality care: (a) enhancing the patient experience of care; (b) creating healthy populations; (c) reducing healthcare costs; (d) improving the care delivery experience; and (e) health equity [23]. Gaps in previous intervention designs include lack of emphasis on patient experience of care and considerations for health equity [23].

The Aging, Community and Health Research Unit – Community Partnership Program (ACHRU-CPP) is a 6-month self-management intervention for community-living older adults (≥ 65 years old), diagnosed with Type 1 or Type 2 diabetes and at least one other chronic condition, and their family/friend caregivers (≥ 18 years old). The intervention was evaluated in a feasibility study in Ontario, Canada [24], followed by a clinical trial in selected primary care and community settings in two Canadian provinces (Ontario and Alberta) [25-27]. A multi-jurisdictional pragmatic RCT is currently in progress to evaluate the implementation and effectiveness of the ACHRU-CPP in three Canadian provinces. To better understand how to

For peer review only - http://bmjopen.bmj.com/site/about/guidelines.xhtml

address the complex needs of older adults, we sought to assess the experiences and perceived impacts of the ACHRU-CPP from the perspectives of older adults with diabetes and MCC.

Methods

Design

This qualitative study is embedded within the multi-site implementation-effectiveness type II hybrid RCT, as outlined in the protocol paper [28]. This study used a qualitative descriptive design, as described by Sandelowski [29, 30], to provide a fulsome summary while remaining close to the words of participants when describing their experiences with the ACHRU-CPP and its perceived impacts.

ACHRU-CPP Program

The ACHRU-CPP is delivered by an interprofessional team of primary care providers, which includes a Registered Nurse (RN) and Registered Dietitian (RD) or Nutritionist from a primary care setting, and a Program Coordinator (e.g., Registered Kinesiologist) from a local community partner organization (hereafter referred to as the intervention team). Table 1 summarizes the core components of the ACHRU-CPP. Due to the COVID-19 pandemic some participants received virtual visits by phone or videoconferencing. A comparison of results from the virtual and in-person approaches will be published in a future paper.

Table 1. The ACHRU-CPP five core components

Intervention Components	Goals
1. Home/virtual visits (up to 3 home visits) and unlimited follow-up phone calls by a RN and/or RD or Nutritionist	To assess older adults' and caregivers' needs and goals using standardized tools to support a coordinated care plan
2. Monthly group wellness sessions (up to 6 sessions) at a local community centre led by	To provide older adults and caregivers with gentle progressive physical activity, self-

For peer review only - http://bmjopen.bmj.com/site/about/guidelines.xhtml

Intervention Components	Goals
the RN, RD or Nutritionist and Community Program Coordinator	management education for diabetes and other chronic conditions, and healthy lunches and snacks
3. Monthly team case conferences which include a RN, RD or Nutritionist, and Community Program Coordinator	To discuss the health and social care needs of older adults and caregivers, develop and revise the coordinated care plan, and plan topics for group wellness sessions
4. Collaboration with the primary care interprofessional team and other specialists (e.g., family physicians, nurse practitioners, kinesiologists, social workers, home care and social service providers, pharmacists, endocrinologists)	To support primary care and community providers in working collaboratively to develop care plans for older adults, and connect older adults and caregivers to specialists and community resources
5. Nurse-led care coordination/system navigation	To facilitate linkages to other primary healthcare providers, specialists and community care services for older adults and caregivers

Note. RN = Registered Nurse; RD = Registered Dietitian

Patient and Public Involvement

The need for the program was originally identified by community-dwelling older adults with diabetes and other chronic conditions and their caregivers and was subsequently co-designed by older adults in collaboration with primary and community care providers and researchers [24]. In the current RCT, patient partners from the pan-Canadian Steering Committee were involved in reviewing research questions and advising the research team on the selection of outcome measures [28]. Patient and public research partners also participated in local Community Advisory Boards in each site to inform further adaptations to the intervention and support local implementation. Patient and public research partners from the local community advisory boards (RB, GG, LG, CL, DL, AM) and the Steering Committee (LM, FT) also provided input into development of this manuscript by reviewing and interpreting the results and helping to shape the key messages.

For peer review only - http://bmjopen.bmj.com/site/about/guidelines.xhtml

BMJ Open

Setting

The study was conducted in two sites in Ontario, Quebec, and Prince Edward Island, Canada. Each of the six sites was selected to ensure variability in geographic setting (urban and rural); socio-demographic and cultural backgrounds; language spoken (English or French); demonstrated support for the ACHRU-CPP; and the presence of staff to support intervention implementation. An RN and RD or Nutritionist from a primary care setting or diabetes education program worked in partnership with a program coordinator from a local community partner site (e.g., YMCA) to implement the program.

Sample and Recruitment

Older adults were screened for eligibility to participate in the RCT by a trained staff member of the primary care site, as described in the study protocol [28]. Eligible patients met the following inclusion criteria: (a) aged 65 years or older; (b) diagnosis of Type 1 or Type 2 diabetes with at least one other chronic condition; (c) receiving primary care services from one of the participating primary care settings; (d) living within the area served by the primary care setting and community site; (e) able to provide informed consent or has a substitute decision-maker able to provide informed consent on the patient's behalf; and (f) competent in English or French, or has an interpreter competent in English or French.

Following the completion of baseline interviews, patients were randomized to receive the intervention (i.e., ACHRU-CPP) in addition to usual care or usual care alone. A total of 8-10 older adults per site who completed the 6-month intervention were invited to participate in follow-up telephone interviews. Trained research assistants (MY, RC), with no prior relationship with participants, used a telephone script to call selected older adults within two weeks of completing the ACHRU-CPP, to invite them to participate in a telephone interview. Maximum

For peer review only - http://bmjopen.bmj.com/site/about/guidelines.xhtml

BMJ Open

variation purposive sampling [31] was used to select a diverse sample of participants across all 6 sites based on their sex, annual income, ethnicity, and level of participation in all components of the study.

Data Collection

Semi-structured post-intervention telephone interviews were conducted between April 2020 and August 2021. Trained research assistants conducted audio-recorded interviews, ranging from 20 to 60 minutes in length, in English or French. Interviews were transcribed verbatim by experienced transcriptionists. Interviews conducted in English were transcribed and cleaned by trained research staff, while interviews conducted in French were transcribed and translated into English by professional transcriptionists and later validated by a bilingual member of the research team. Transcripts were not returned to participants for their review. The interview guide was created based on: (a) a review of the literature of health and social needs of older adults and caregivers, patient-provider communication, and system navigation and (b) feedback from patient partners and the research team with expertise in aging, community-based supports for older adults and caregivers, and qualitative research. Table 2 provides sample interview questions.

Table 2. Sample interview questions for older adults

**Questions for Older Adults**
1. What did you need the most in the past six months (e.g., physical, emotional, mental or psychological support, transportation, financial assistance, housekeeping, personal care support)?
2. When [name of nurse and dietitian] visited you what types of things did they do during those visits?

For peer review only - http://bmjopen.bmj.com/site/about/guidelines.xhtml

3. For other people who are living with diabetes and other chronic conditions, would you recommend that a nurse or dietitian, such as [name of nurse and dietitian], visit the person at home, make phone calls or both?
4. What types of things did you do at the monthly wellness sessions or during the individual calls with [name of the community program coordinator]?
5. How, if at all, did [name of nurse and dietitian] involve you in decisions about your care?
6. How, if at all, did [name of nurse and dietitian] help you to connect with other community health or social services to help you?
7. To what extent did the nurse and dietitian help to address your needs or the issues that were most important to you?
8. How happy are you with the overall care that you received from [name of nurse and dietitian]?
9. Was the information given by [name of nurse and dietitian] and other health professionals about care consistent (across individuals)?
10. Is there anything else about your experiences with [name of nurse and dietitian] that you would like to add that we haven't already discussed?

Data Analysis

Themes were generated using the Braun and Clarke's experiential thematic analysis framework [32] and organized under relevant constructs of the Consolidated Framework for Implementation Research [33]. Thematic analysis was selected to ensure that the development of themes was informed by the experiences and perceived intervention benefits of older adults. The six phases of thematic analysis include: (a) becoming familiar with the data; (b) coding; (c) developing themes; (d) reviewing themes; (e) constructing a definition for themes and labelling them; and (f) creating a report [32]. A female research assistant with doctoral level training in qualitative research (MY) used the data management software NVivo version 12 [34] to perform coding. MY is fluent in both languages and coded in English. Results were shared with the team

For peer review only - http://bmjopen.bmj.com/site/about/guidelines.xhtml

in English only. Following the creation of codes, these were further examined for patterns to generate themes. Themes were shared with the research team, including patient partners, to ensure they were reflective of the data.

Rigour and Trustworthiness

Consensus was reached by all authors prior to the inclusion of themes in the final report. Lincoln and Guba's validation criteria [35] were applied in this study to enhance the study's rigour. To support the credibility of findings, investigator triangulation was used in data analysis through team meetings with 5-7 members to review the coding structure and evidence of themes. These members included patient and public research partners and researchers of various disciplines with expertise in qualitative research, gerontology, and community-based interventions. Conflicts were resolved through team consensus. To facilitate transferability of findings, the study sample and setting were described in detail. To support dependability and confirmability of findings, the research team kept an audit trail of study processes.

Results

A total of 295 older adults were enrolled in the RCT and randomly allocated to receive the ACHRU-CPP or usual care. At the time of data collection, 53 older adults who had completed the 6-month intervention were approached to participate in the qualitative interviews and 45 accepted (84.9%). The rate of acceptance by site was as follows: Site 1, 100% (10/10); Site 2, 89.9% (8/9); Site 3, 89.9% (8/9); Site 4, 60% (6/10); Site 5, 100% (8/8); and Site 6, 71.4% (5/7). Out of the 45 participants, there was good uptake of home visits with a mean of 3.1 (SD (standard deviation) = 1.5) and group wellness sessions with a mean of 2.7 (SD = 1.9). Where the providers deemed clinically necessary participants received more than the allotted 3

For peer review only - http://bmjopen.bmj.com/site/about/guidelines.xhtml

BMJ Open

home visits. Competing commitments such as doctor appointments and lack of interest were barriers to attending group wellness sessions for some participants.

Demographic Characteristics

The mean age of the 45 older adults who participated in interviews was 71.1 years and the mean length of time living with diabetes was 18.8 years (SD = 10.6). Most were female (55.6%), retired from paid work (80%), had Type 2 diabetes (93.3%), and reported 4-6 chronic conditions (44.4%). Hypertension, hyperlipidemia, and osteoarthritis and other arthritis (e.g., rheumatoid arthritis) were the most reported chronic conditions. Table 3 summarizes demographic characteristics of participants.

Table 3. Demographic characteristics of interview participants

**Older Adults (n=45)**	
**Category**	**n (%)**
Age (mean [Standard Deviation]): 71.7 [6.5]	
65-70	26 (57.8)
71-75	7 (15.6)
76+	12 (26.7)
Sex	
Female	25 (55.6)
Male	20 (44.4)
Marital Status	
Married or living with a partner	21 (46.7)
Divorced, never married, separated, or widowed	23 (51.1)
Refused	1 (2.2)
Highest Level of Education	
Completed a graduate or professional degree	6 (13.3)
Completed a bachelor’s degree	10 (22.2)
Had some university education or completed a community college, technical college, or postsecondary program	12 (26.7)
Completed secondary school	10 (22.2)
Did not complete secondary school	7 (15.6)
Current Employment Status	

For peer review only

For peer review only - http://bmjopen.bmj.com/site/about/guidelines.xhtml

Older Adults (n=45)	
**Category**	**n (%)**
Retired from paid work	36 (80.0)
Employed full-time	4 (8.9)
Employed part-time	2 (4.4)
Unemployed and looking for work	1 (2.2)
Refused	2 (4.4)
Annual Household Income	
$150,000 or more	2 (4.4)
$100,000 or more, but less than $150,000	2 (4.4)
$50,000 or more, but less than $100,000	12 (26.7)
$20,000 or more, but less than $50,000	16 (35.6)
Less than $20,000	11 (24.4)
Refused	2 (4.4)
Born in Canada	
Yes	31 (68.9)
Ethnic/Racial Group	
White/Caucasian	32 (71.1)
Black	3 (6.7)
Caribbean/Guyanese	3 (6.7)
Filipino	2 (4.4)
First Nations	1 (2.2)
South Asian	1 (2.2)
Southeast Asian	1 (2.2)
Chinese	1 (2.2)
Japanese	1 (2.2)
Language(s) Spoken	
English	37 (82.2)
French	15 (33.3)
Living with Others (e.g., spouse, children, other relative, friend, group setting)	
Yes	27 (60.0)
Type of Diabetes	
Type 1 diabetes	1 (2.2)
Type 2 diabetes	42 (93.3)
Unknown	2 (4.4)
Number of Chronic Conditions (mean [Standard Deviation]): 5.6 [2.9]	
1-3	11 (24.4)
4-6	20 (44.4)
7-9	8 (17.8)
10 +	6 (13.3)
Commonly Reported Chronic Conditions	
Hypertension	34 (75.6)
Hyperlipidemia	27 (60.0)
Osteoarthritis and other arthritis	18 (40.0)
Cardiovascular disease	16 (35.6)

For peer review only - http://bmjopen.bmj.com/site/about/guidelines.xhtml

Older Adults (n=45)	
**Category**	**n (%)**
At Least 1 Emergency Room Visit in the Last 6 Months	
6 months prior to ACHRU-CPP	8 (17.8)
6-month follow-up	7 (15.6)

Themes

Themes were grouped into two categories, experiences, and perceived impacts of the ACHRU-CPP. Table 4 provides an overview of themes. The words in italics that label the theme are taken verbatim from transcripts. Similarly, participant quotes in the narrative that follows are noted in italics and identified by OA for older adult, # for site number, and ### for participant number.

Table 4. Themes of older adult experiences and perceived impacts with the ACHRU-CPP

**Experiences with the ACHRU-CPP**
• In-depth dialogue with *"professional friends"*<br>• Socialized with *"people with the same type of health problems"*<br>• Person-centred care by *"more than one knowledgeable person"*<br>• Ongoing contact with providers so *"you are not alone"*<br>• Need to address ethnic/cultural differences through a *"personal session"*
**Perceived Impacts of the ACHRU-CPP**
• Improved diabetes self-management behaviours: *"make more proactive steps"*<br>• Added connection to health and social support services *"that could help me"*

For peer review only - http://bmjopen.bmj.com/site/about/guidelines.xhtml

For peer review only

BMJ Open

Experiences with the ACHRU-CPP

Overall, older adults reported positive experiences with the ACHRU-CPP. They experienced: (a) in-depth dialogue with "*professional friends"*; (b) socialized with *"people with the same type of health problems"*; (c) person-centred care by *"more than one knowledgeable person"; (d)* ongoing contact with providers so *"you are not alone";* and identified the (e) need to address ethnic/cultural differences: *"eating has to do with seasons".*

In-depth dialogue with *"professional friends".* In-person home and virtual visits were perceived by older adults as more relaxed compared to clinic visits and provided opportunities for in-depth dialogue about health and social issues with providers. "*The home visits are more relaxed, if you were at a clinic, you got a time slot you got to meet whatever is transacted in that timeframe"* (OA_1_152). Findings revealed that older adults had a lot of concerns and questions regarding diabetes and other chronic conditions that were often left unanswered by providers due to the nature of clinic visits that are limited by time and ailment. Home visits helped to build trust between older adults and the providers, which facilitated the exploration of concerns and needs beyond diabetes such as safe housing and transportation issues. "*I felt I could trust her [nutritionist]*" (OA_5_037). Providers were approachable and understanding of older adults' situations and were considered as friends and confidants. *"They [providers] were professional friends"* (OA_2_242). Their approach was especially important when discussing sensitive topics such as mental health concerns. "*I had my sick niece calling me and that was stressing me, so she [nurse] said to "Let go. When we are stressed, that's not always good". So how to manage my stress"* (OA_6_023).

For peer review only - http://bmjopen.bmj.com/site/about/guidelines.xhtml

BMJ Open

Socialized with *“people with the same type of health problems”*. Older adults and caregivers perceived that group wellness sessions helped them meet others who understood what it is like to live with diabetes and other chronic conditions. *“The fact of socializing with other people with the same types of health problems as we do”* (OA_5_037). Learning about the burden that others experience with their conditions encouraged older adults to support each other and express their frustrations. The sessions provided opportunities for group exercises which provided peer motivation. The group sessions were particularly helpful for older adults who were socially isolated, and some older adults became friends because of the sessions.

Person-centred care by *“more than one knowledgeable person”*. Older adults appreciated that they received person-centred care from a team of providers through the ACHRU-CPP to discuss diabetes, their other chronic conditions, and social concerns. Some older adults perceived that in usual care providers at times delivered care in silos or independent of other disciplines. Older adults valued providers working collaboratively to meet their needs. *“It was good that they worked in a team. More than one knowledgeable person. That was important”* (OA_2_242). They felt that providers were listening to their concerns and that, prior to meeting with the intervention team, it was difficult to find the right person to talk to about diabetes.

> *I enjoyed having them come to visit. I don’t talk to a lot of people about my diabetes because I don’t feel it’s that complicated, but nobody really wants to listen about your health issues.* (OA_3_032)

Providers supported the management of other conditions in addition to diabetes. *“I was having troubles with my bowels, but we got that regulated and it’s good”* (OA_4_075).

Ongoing contact with providers so *“you are not alone”*. Providers made older adults feel that someone was concerned about their well-being. “*It’s not as if we are just left alone with*

For peer review only - http://bmjopen.bmj.com/site/about/guidelines.xhtml

our problems. What you are doing is very good; continue" (OA_5_128). Managing diabetes and MCC felt burdensome for older adults, and they appreciated regular contact with providers. Follow-up phone calls were well received by older adults, especially by those living alone or with little support, and ensured that they "*haven't fallen through the cracks"* (OA_3_058). The ongoing follow-up with the team reinforced familiar information that older adults had forgotten to put into practice over time. A few participants reported that once the intervention stopped at the 6-month period they felt that there was a break in the social connection with peers and formal providers.

Need to address ethnic/cultural differences through a *"personal session".* Some ethnic groups may have language barriers and be *"very shy and they don't approach people unless someone else pushes them to go [join programs]"* (OA_1_061). Some older adults perceived that individual wellness sessions with providers may be helpful for those with language barriers. *"Some of them had a bit of a language problem. I think a personal session would be much more helpful"* (OA_2_086). The following challenge experienced by one participant also exemplifies the need to provide dedicated time, encouragement and support for older adults to share their personal cultural practices during interactions with peers and providers.

> *One of the things I learned about myself from my community [Indigenous community] and my family is that eating has to do with seasons...Your year-round diet has to do with what's available to you...I mentioned that one time in the group [Group Wellness Sessions]and they thought that had nothing to do with what the topics were.* (OA_2_013)

Providing opportunities to discuss cultural practices promotes diversity within groups and learning across cultures.

Perceived Impacts

For peer review only - http://bmjopen.bmj.com/site/about/guidelines.xhtml

Older adults perceived that the ACHRU-CPP had positive impacts on their health and well-being as a result of: (a) improved diabetes self-management behaviours: *"make more proactive steps"* and (b) added connection to health and social support services *"that could help me"*.

Improved diabetes self-management behaviours: *"make more proactive steps".* Older adults felt that the ACHRU-CPP helped them to recognize and take more action in preventing complications, such as hypoglycemia and hyperglycemia, and decreasing their blood pressure, as described in the following quote. They recognized that reaching a level of effective self-management of chronic conditions can be complicated and they appreciated the support provided through the ACHRU-CPP.

> *What [the nurse] and [dietitian] caused me to be concerned about is to make more proactive steps, to watch out for those low blood sugars. I really didn't realize how badly they could affect you. Shaking and double vision is one thing but not being able to drive, that's quite another thing*. (OA_2_242)

Older adults indicated that they gained nutrition knowledge by participating in the ACHRU-CPP. "*I improved it [eating habits]. I had to eat more fruit and vegetables...and after that, I had to hydrate myself more and add more fibre to my diet"* (OA_5_027). Older adults perceived that changes made to their diet could lead to multiple benefits including weight loss and decreased sugar levels. "*...drinking more water and diet, I think that's what was important, and I lost weight at the same time. By eating well, fewer treats, being more careful, the sugar levels were lower"* (OA_6_005).

Older adults perceived they were able to build more muscle mass and lose weight and experienced less difficulty in climbing stairs. Some older adults felt that they were not exercising enough prior to participating in the ACHRU-CPP and perceived that the providers helped them to meet their activity goals.

For peer review only - http://bmjopen.bmj.com/site/about/guidelines.xhtml

They really helped me with the exercise piece. I had poor balance. It was the [nurse and dietitian] that really said 'why don't you try doing this? I'll give you a call this week and see if you got out to do your walk', and then I'd promise them that I would start journaling my steps so little by little I started increasing my exercise. (OA_2_247)

Added connection to health and social support services *"that could help me"*. Older adults indicated that they were referred to and connected with health and social support services (e.g., food bank, exercise program, smoking cessation, home care, social work, arts program). Living well with diabetes and other chronic conditions was perceived by older adults to require more than just medical care. Some older adults required supports to meet their basic needs, as they were not able to afford groceries or travel far distances for groceries and medications. "*She [nurse] referred me [for medication delivery]*" (OA_1_061). Some older adults required mental health support to enhance their ability to manage diabetes and other chronic conditions. The intervention team followed-up with older adults after making referrals to ensure that they were connected. Older adults were referred to local community resources that offered free or low-cost services. "*They told me I could go to [name of community centre] to do exercise"* (OA_4_016). Prior to participating in the ACHRU-CPP older adults indicated that they had seldom been referred to programs outside of the clinic and therefore they were not aware of available community resources.

Older adults found it important to be aware of publicly-funded resources, such as tax rebates and housing options, in case they or their loved one required these in the future. By attending group wellness sessions, they learned about the types of programs the community partner sites had to offer. *"They [community partner site] have virtual classes and they're all free. They have special classes just for seniors"* (OA_2_013). Despite the mostly positive impacts of being connected with relevant health and social services, some participants reported that not all of their needs were met at the group wellness sessions. For example, some did not

For peer review only - http://bmjopen.bmj.com/site/about/guidelines.xhtml

qualify for certain financial assistance programs. *"They just said you have to qualify [for financial assistance] because...you have to apply for this, you have to apply for this"* (OA_1_058). Some participants reported that it was difficult to absorb all of the information presented and not all of the information was relevant to their situation. It was challenging to tailor group wellness sessions to individual needs compared to home visits which allowed further individualized tailoring.

Discussion

Key findings of this study were that the ACHRU-CPP increased in-depth dialogue with '*professional friends'* and provided person-centred care and ongoing contact with providers to prevent feelings of being alone. The use of a social determinants of health approach by the intervention teams was a novel component of the program and highlighted the importance of addressing social aspects of care for older adults with diabetes and MCC. Group interactions brought together participants with the same type of health issues and provided peer motivation and support. Participants identified that the program would benefit from adaptations to address cultural and language differences among older adults living with diabetes and other conditions in Canada.

Older adults with multimorbidity and diabetes face significant burden in managing their chronic conditions, which challenge their self-care and adversely affect their overall quality of life [36]. In addition to managing diabetes symptoms and complications (e.g., hypoglycemia and hyperglycemia, visual impairment, neuropathy) which negatively impact their physical functioning, older adults with diabetes often experience psychological burden associated with complex medication and diet regimens (e.g., medication and insulin management and glycemic

For peer review only

For peer review only - http://bmjopen.bmj.com/site/about/guidelines.xhtml

control) [36]. Added pressures include financial burden, food insecurity, social isolation, lack of social support, as well as frailty, and the burden of comorbid chronic conditions, e.g., anxiety, depression [36]. Several of these challenges were experienced by our study participants. The largely positive response to the program could be attributed to the support that participants received in managing the burden associated with living with diabetes and other chronic conditions. High quality care for this population to prevent diabetes distress (i.e., challenges faced when dealing with the demands of diabetes), requires good communication and trusting relationships with providers, social and peer support, and self-management education [37].

A novel finding of this study was that the ACHRU-CPP was perceived by older adults from three Canadian provinces to positively impact their self-management practices of diabetes and MCC by helping to address their broad health and social needs. This has not been documented before in similar studies [19, 20]. This may be because the ACHRU-CPP was longer and more person-centred compared to other interventions. Interventionists were able to directly assess the home context and understand the impacts of social determinants of health. Mental health concerns and lack of support, which were experienced by our study participants, can impact the ability of older adults to effectively manage diabetes and lead to severe hypoglycemia, elevated HbA1c levels, a greater number of missed insulin doses, and a higher risk for diabetic ketoacidosis and mortality [38, 39, 3].

When healthcare providers recommend lifestyle changes, they need to recognize that social determinants of health such as housing, food security, social relationships, and financial stability have an impact on older adults' abilities to enact them [40]. In the current study, the intervention team assessed the social determinants of health and found ways to address them, such as by linking older adults with relevant community resources, to help overcome barriers to

For peer review only - http://bmjopen.bmj.com/site/about/guidelines.xhtml

self-management. The intervention team targeted health literacy of older adults and caregivers through education, capacity building, and opportunities for dialogue among peers and experts.

In the current study, older adults appreciated receiving person-centred care supported by a team of providers from primary care and community sectors and the engagement of providers outside of the intervention team (such as social workers). Due to the complex nature of diabetes and MCC, interprofessional collaboration has been found to lead to positive outcomes for persons with Type 2 diabetes, such as improvements in HbA1c levels, regular testing of blood glucose levels, and smoking cessation [41, 42].

What is unique about this study is the partnership between healthcare providers and a Program Coordinator from a local community partner site. These health and social services can be underutilized if healthcare providers are not aware of them. As per the Quintuple Aim [23] there is a need to optimize the use of existing community-based services for patients, address any barriers to accessing these services, and for strong coordination of services [43].

Person-centred care was perceived to be key strength of the ACHRU-CPP that enabled older adults to improve self-management practices related to diabetes and MCC. In working towards a person-centred learning health system, defined as a health system that integrates internal data, patient experience, and research evidence [44], the priorities and experiences of older adults should be regularly reported in data systems so that services that meet their needs are developed and evaluated as part of continuous quality improvement processes [38]. As seen in this study, patient experience can be improved by having a provider connect patients with other interdisciplinary health and social care providers to ensure that smooth transitions between services occur [45].

For peer review only - http://bmjopen.bmj.com/site/about/guidelines.xhtml

In practice and policy, there is a need for integrated care delivery models that leverage community partnerships to help fill gaps in meeting the complex health and social needs of older adults with diabetes. To advance Quintuple Aim outcomes [23], it is critical to assess patient experiences with healthcare services as part of intervention research and practice to improve health system performance.

The strengths of the study include its rigorous qualitative design and large sample size, and the inclusion of diverse participants with regards to sex, marital status, and annual income, from multiple sites across Canada. A limitation of the study was related to the sample as there was a lack of cultural diversity and under-representation of older adults from marginalized communities. A Diabetes Canada roundtable of key stakeholders recently emphasized the need to implement community-based interventions [46], such as the ACHRU-CPP, to better support older adults with diabetes and MCC living in marginalized communities. The need for community-based interventions is based on the premise that racial and socioeconomic disparities disproportionally affect marginalized older adults with diabetes and MCC and put them at an increased risk for diabetes complications and mortality [47].

Conclusion

Overall, older adults with diabetes and MCC reported a positive experience and felt that the ACHRU-CPP had a positive impact in supporting diabetes self-management. Study findings reveal the need to ensure that older adults receive ongoing support and contact with a collaborative team of primary care and community providers to better meet the complex needs associated with daily self-management of diabetes and MCC. Results also shine light on the broader social context that constitutes the life world of older adults and how chronic disease self-

For peer review only

For peer review only - http://bmjopen.bmj.com/site/about/guidelines.xhtml

management interventions need to address these contexts comprehensively through tailoring to individual circumstances. It is our hope that these findings will help usher in a new era of contextually informed person-centred care.

Acknowledgements

We thank the older adults and caregivers who participated in this study, as well as the nurses, dietitians, nutritionists and community program coordinators who provided the intervention. We also thank the managers of intervention teams, the recruiters, research assistants, and the study sites for their support of this study. Thanks to Robyn Connors for conducting interviews with study participants. We thank the research team in the Aging, Community and Health Research Unit (https://achru.mcmaster.ca/) for supporting this study. We would also like to thank the Community Advisory Board members, including people with lived experience with diabetes, who supported local implementation of the project in each province.

We acknowledge the following authors who are members of the **ACHRU-CPP Research Team**: **Jenny Ploeg**, School of Nursing, Faculty of Health Sciences, McMaster University, Hamilton, Ontario, Canada; **Maureen Markle-Reid**, School of Nursing, Faculty of Health Sciences, McMaster University, Hamilton, Ontario, Canada; **Ruta Valaitis**, School of Nursing, Faculty of Health Sciences, McMaster University, Hamilton, Ontario, Canada; **Kathryn Fisher,** School of Nursing, Aging, Community and Health Research Unit, Faculty of Health Sciences, McMaster University, Hamilton, Ontario, Canada; **Rebecca Ganann**, School of Nursing, Aging, Community and Health Research Unit, Faculty of Health Sciences, McMaster University, Hamilton, Ontario, Canada; **Johanne Blais**, Department of Family Medicine and Emergency Medicine, Faculty of Medicine, Université Laval, Quebec City, Quebec; **Andrea**

For peer review only - http://bmjopen.bmj.com/site/about/guidelines.xhtml

Gruneir, Department of Family Medicine Research Program, University of Alberta, Edmonton, Alberta, Canada; **France Légaré**, VITAM-Centre de recherche en santé durable, Université Laval, Quebec City, Quebec, Canada; **Janet MacIntyre**, Faculty of Nursing, University of Prince Edward Island, Charlottetown, Prince Edward Island, Canada; **William Montelpare**, Department of Applied Human Sciences, Faculty of Science, University of Prince Edward Island, Prince Edward Island, Canada; **Jean-Sébastien Paquette**, Department of Family Medicine and Emergency Medicine, Faculty of Medicine Université Laval, Québec, Canada; **Marie-Eve Poitras**, Department of Family Medicine and Emergency Medicine, Faculty of Medicine and Health Sciences, Université de Sherbrooke Chicoutimi, Quebec, Canada; **Angela Riveroll**, Department of Applied Human Sciences, Faculty of Science, University of Prince Edward Island, Charlottetown, Prince Edward Island, Canada, **Ali Ben Charif,** CubecXpert, Quebec City, Quebec, Canada; **Dean Eurich**, School of Public Health, University of Alberta, Edmonton, Alberta, Canada; **Amiram Gafni**, Department of Health Research Methods, Evidence and Impact, Faculty of Health Sciences, McMaster University, Hamilton, Ontario, Canada; **Gary Lewis**, Department of Medicine and Department of Physiology, University of Toronto, Toronto, Ontario, Canada; **Lynne Mansell**, Patient/Public Research Partner, Alberta, Canada; **Melissa Northwood**, School of Nursing, Aging, Community and Health Research Unit, Faculty of Health Sciences, McMaster University, Hamilton, Ontario, Canada; **Janet Pritchard**, Interdisciplinary Science and Kinesiology, Faculty of Science, McMaster University, Hamilton, Ontario, Canada; **Cheryl Sadowski**, Faculty of Pharmacy and Pharmaceutical Sciences, University of Alberta, Edmonton, Alberta, Canada; **Diana Sherifali**, School of Nursing, Faculty of Health Sciences, McMaster University, Hamilton, Ontario, Canada; **Frank Tang**, Patient/Public Research Partner, Ontario, Canada**; Lehana Thabane,** Department of Health

For peer review only - http://bmjopen.bmj.com/site/about/guidelines.xhtml

Research Methods, Evidence and Impact, Faculty of Health Sciences, McMaster University, Hamilton, Ontario, Canada; **Ross Upshur**, Bridgepoint Active Healthcare, Toronto, Ontario, Canada; **Tyler Williamson**, Centre for Health Informatics, Cumming School of Medicine and Department of Community Health Sciences, University of Calgary, Calgary, Alberta, Canada; **Marie-Lee Yous,** School of Nursing, Faculty of Health Sciences, McMaster University, Hamilton, Ontario, Canada.

For peer review only

Author Contributions

Conceptualization: JP, MMR, RV, KF, RG, FL, WM.

Formal Analysis: MY, RG, TC, JP.

Funding Acquisition: JP, MMR, RV, KF, RG, AG, FL, JM, WM.

Investigation: JP, MMR, RV, KF, RG, TC, FL, WM, MY.

Methodology: JP, MMR, RV, KF, RG, MY, FL, WM.

Project Administration: JP, MMR, RV, TC, FL, WM.

Resources: JP, MMR, RV, FL, WM.

Supervision: JP, MMR, RV, RG, TC, FL, WM.

Validation: MY, JP, MMR, RV, KF, RG, TC, RC.

Writing – Original Draft: MY.

Writing – Review and Editing: MY, RG, TC, JP, MMR, RV, KF, FL, JM, WM. The authors read and approved the final manuscript.

Funding

For peer review only - http://bmjopen.bmj.com/site/about/guidelines.xhtml

This study is supported, in part, by funding from the Canadian Institutes of Health Research Strategy for Patient-Oriented Research (SPOR) Primary and Integrated Health Care Innovations Network: Programmatic Grants (Funding Reference Number: KPG-156883) in partnership with: Diabetes Action Canada, a Canadian Institutes for Health Research (CIHR) Strategy for Patient-Oriented Research Network in Chronic Disease (project reference #1.1.1ACHR); McMaster Institute for Research on Aging (Hamilton, ON); McMaster University School of Nursing; Réseau-1 Québec; Fonds de Recherche du Québec (FRQS); Scarborough Health Network Foundation. This research was also undertaken, in part, thanks to the funding from Dr. Markle-Reid's Tier 2 CIHR Canada Research Chair. The study was investigator-initiated. The funders of this study had no role in study design, data collection, data analysis, data interpretation or writing the manuscript.

Competing Interests

None declared.

Availability of Data and Materials

The data for this research consists of questionnaires and interviews. Raw data such as audio-files and interview transcripts cannot be publicly released due to the risk of compromising participant confidentiality related to identification of voices and publicly exposing personal information.

Ethics Statement

For peer review only - http://bmjopen.bmj.com/site/about/guidelines.xhtml

Institutional ethics approval was obtained from the following: the Hamilton Integrated Research Ethics Board (#5101); the Scarborough Health Network Research Ethics Board (#NEP-18-014); the Unity Health Toronto Research Ethics Board (#18-336); University of Prince Edward Island Research Ethics Board (#6008019); Prince Edward Island Research Ethics Board (#6008019); and Centre intégré universitaire de santé et de services sociaux (CIUSSS) de la Capitale-Nationale (MP-13-2019-1670). Trained research assistants obtained informed consent from all older adult participants prior to their participation in the study, and all participants received a copy of their consent form, in person or by mail. All participants gave permission to audio-record their interview. All participant information was kept confidential in a secure location (e.g., locked cabinet in a secured office, password-protected encrypted electronic folders), and data were de-identified using unique study IDs. Participants who completed a qualitative interview received a $25.00 gift card as an honorarium.

For peer review only

For peer review only - http://bmjopen.bmj.com/site/about/guidelines.xhtml

References

[1] World Health Organization. Diabetes. Available from: https://www.who.int/en/news-room/fact-sheets/detail/diabetes. 2022. Accessed 3 Jan 2022.
[2] Kirkman MS, Briscoe VJ, Clark N, et al. Diabetes in older adults: A consensus report. *J Am Geriatr Soc* 2012;35(12):2650-64.
[3] Dhaliwal R, Weinstock RS. Management of type 1 diabetes in older adults. *Diabetes Spectr*. 2014;27(1):9-20.
[4] Sinnige J, Braspenning J, Schellevis F, et al. The prevalence of disease clusters in older adults with multiple chronic diseases–a systematic literature review. *PloS one* 2013;8(11):e79641.
[5] Lin PJ, Kent DM, Winn A, et al. Multiple chronic conditions in type 2 diabetes mellitus: prevalence and consequences. *Am J Manag Care* 2015;21(1):e23-34.
[6] Fisher K, Griffith L, Gruneir A, et al. Comorbidity and its relationship with health service use and cost in community-living older adults with diabetes: A population-based study in Ontario, Canada. *Diabetes Res Clin Pract* 2016;122:113-123.
[7] Willi C, Bodenmann P, Ghali WA, et al. Active smoking and the risk of type 2 diabetes: A systematic review and meta-analysis. *JAMA* 2007; 298:2654-64.
[8] Ploeg J, Matthew-Maich N, Fraser K, et al. Managing multiple chronic conditions in the community: A Canadian qualitative study of the experiences of older adults, family caregivers and healthcare providers. *BMC Geriatr* 2017;17.
[9] The Change Foundation. Family caregiver assessment in health care settings, summary of the change foundation's literature review and environment scan project. Toronto, August 2016.
[10] McGilton KS, Vellani S, Yeung L, et al. Identifying and understanding the health and social care needs of older adults with multiple chronic conditions and their caregivers: A scoping review. BMC Geriatr 2018;18(1):1-33.
[11Craig P, Dieppe P, Macintyre S, et al. Developing and evaluating complex interventions: the new Medical Research Council guidance. *BMJ*. 2008;337:1-6.
[12] Whittemore R. A systematic review of the translational research on the diabetes prevention program. *Transl Behav Med* 2011;1:480-91.
[13] Tuomilehto J, Schwarz P, Lindström J. Long-term benefits from lifestyle interventions for type 2 diabetes prevention: Time to expand the efforts. *Diabetes Care* 2011;34:S210-14.
[14] Smith SM, Wallace E, O'Dowd T, et al. Interventions for improving outcomes in patients with multimorbidity in primary care and community settings. *Cochrane Database Syst Rev* 2016;3.
[15] Yoon U, Kwok LL, Magkidis A. Efficacy of lifestyle interventions in reducing diabetes incidence in patients with impaired glucose tolerance: A systematic review of randomized controlled trials. *Metabolism* 2013;62:303-14.
[16] Busetto L, Luijkx KG, Elissen AMJ, et al. Context, mechanisms and outcomes of integrated care for diabetes mellitus type 2: A systematic review. *BMC Health Serv Res* 2016;16:1-14.
[17] Diabetes Prevention Program Research Group. 10-year follow-up of diabetes incidence and weight loss in the diabetes prevention program outcomes study. *Lancet* 2009;374:1677-86.

For peer review only - http://bmjopen.bmj.com/site/about/guidelines.xhtml

[18] Wong KC, Wong FKY, Yeung W, et al. The effect of complex interventions on supporting self-care among community-dwelling older adults: A systematic review and meta-analysis. *Age Ageing* 2017;1-9.
[19] Azami G, Soh KL, Sazlina SG, et al. Effect of a nurse-led diabetes self-management education program on glycosylated hemoglobin among adults with type 2 diabetes. *J Diabetes Res* 2018:1-13.
[20] Chow SK, Wong FK. A randomized controlled trial of a nurse-led case management programme for hospital-discharged older adults with co-morbidities. *J Adv Nurs* 2014;70(10):2257-71.
[21] Dahal PK, Hosseinzadeh H. Association of health literacy and diabetes self-management: A systematic review. *Aust J Prim Health* 2020;25(6):526-33.
[22] Ferris R, Blaum C, Kiwak E, et al. Perspectives of patients, clinicians, and health system leaders on changes needed to improve the health care and outcomes of older adults with multiple chronic conditions. *J Aging Health* 2018;30(5):778-99.
[23] Nundy S, Cooper LA, Mate KS. The Quintuple Aim for health care improvement: A new imperative to advance health equity. *JAMA* 2022;327(6):521–522.
[24] Markle-Reid M, Ploeg J, Fisher K, et al. The Aging, Community and Health Research Unit—Community Partnership Program for older adults with type 2 diabetes and multiple chronic conditions: A feasibility study. *Pilot Feasibility Stud* 2016 2(1):24.
[25] Markle-Reid M, Ploeg J, Fraser KD, et al. Community program improves quality of life and self-management in older adults with diabetes mellitus and comorbidity. J Am Geriatr Soc. 2018 Feb;66(2):263-73.
[26] Miklavcic JJ, Fraser KD, Ploeg J, Markle-Reid M, Fisher K, Gafni A, Griffith LE, Hirst S, Sadowski CA, Thabane L, Triscott JA. Effectiveness of a community program for older adults with type 2 diabetes and multimorbidity: A pragmatic randomized controlled trial. BMC Geriatr. 2020 Dec;20(1):1-14.
[27] Fisher K, Markle-Reid M, Ploeg J, Bartholomew A, Griffith LE, Gafni A, Thabane L, Yous ML. Self-management program versus usual care for community-dwelling older adults with multimorbidity: A pragmatic randomized controlled trial in Ontario, Canada. J Comorb. 2020 Oct 14;20(174):1-14.
[28] Ploeg J, Markle-Reid M, Valaitis R, et al. The Aging, Community and Health Research Unit Community Partnership Program for older adults with diabetes and multiple chronic conditions: Study protocol for a randomized controlled trial. *BMC Geriatrics* 2022;22(99):1-22.
[29] Sandelowski M. Whatever happened to qualitative description? Res Nurs health. 2000;23(4):334-40.
[30] Sandelowski M. What's in a name? Qualitative description revisited. Res Nurs Health. 2010 Feb;33(1):77-84.
[31] Patton MQ. Qualitative evaluation and research methods. Beverly Hills, CA: SAGE, 1990.
[32] Braun V, Clarke V. Successful qualitative research: A practical guide for beginners. London, England: SAGE, 2013.
[33] Damschroder LJ, Aron DC, Keith RE, et al. Fostering implementation of health services research findings into practice: A consolidated framework for advancing implementation science. Implement Sci. 2009 Dec;4(1):1-5.
[34] QSR International Pty Ltd. NVivo (Version 12). 2018. Available from: https://www.qsrinternational.com/nvivo-qualitative-data-analysis-software/home
[35] Lincoln YS, Guba EG. Naturalistic inquiry. Sage Publications, 1985.

For peer review only - http://bmjopen.bmj.com/site/about/guidelines.xhtml

[36] Sayyed Kassem L, Aron DC. The assessment and management of quality of life of older adults with diabetes mellitus. Expert Rev Endocrinol Metab. 2020 Mar 3;15(2):71-81.
[37] Skinner TC, Joensen L, Parkin T. Twenty-five years of diabetes distress research. Diab Med. 2020 Mar;37(3):393-400.
[38] Robinson DJ, Coons M, Haensel H, et al. Diabetes and mental health. *Can J Diabetes* 2018 Apr 1;42:S130-41.
[39] Lynch CP, Gebregziabher M, Zhao Y, et al. Impact of medical and psychiatric multi-morbidity on mortality in diabetes: emerging evidence. *BMC Endocr Disord* 2014;14(1):1-8.
[40] Kuluski K, Guilcher SJ. Toward a person-centred learning health system: Understanding value from the perspectives of patients and caregivers. *Healthc Pap* 2019;18(4):36-46.
[41] Hellquist K, Bradley R, Grambart S, et al. Collaborative practice benefits patients: An examination of interprofessional approaches to diabetes care. *Health Interprofessional Pract* 2012;1(eP1017).
[42] O'Connor PJ, Desai J, Solberg LI, et al. Randomized trial of quality improvement intervention to improve diabetes care in primary care settings. *Diabetes Care* 2005;28,1890–97.
[43] Valaitis RK, Wong ST, MacDonald M, et al. Addressing quadruple aims through primary care and public health collaboration: ten Canadian case studies. BMC Public Health 2020;20(1):1-6.
[44] Agency for Healthcare Research and Quality. About learning health systems. 2019. Available from: https://www.ahrq.gov/learning-health-systems/about.html. Accessed 12 Feb 2022.
[45] Rapid Improvement Support and Exchange. RISE brief 1. OHT (Ontario Health Team) building blocks. 2019. Available from: https://www.mcmasterforum.org/docs/default-source/rise-docs/rise-briefs/rb1_oht-building-blocks.pdf?sfvrsn=71b154d5_27. Accessed 12 Feb 2022.
[46] Diabetes Canada. Summary of Diabetes Canada Diabetes 360˚ Ontario roundtable. 2019. Available from: https://www.diabetes.ca/DiabetesCanadaWebsite/media/Advocacy-and-Policy/Advocacy%20Reports/Ontario-Diabetes-360-Strategy_Roundtable-Summary_FINAL.pdf. Accessed 12 Feb 2022.
[47] Clements JM, West BT, Yaker Z, et al. Disparities in diabetes-related multiple chronic conditions and mortality: The influence of race. *Diabetes Res Clin Pract* 2020;159(107984):1-19.

For peer review only - http://bmjopen.bmj.com/site/about/guidelines.xhtml

COREQ (COnsolidated criteria for REporting Qualitative research) Checklist

A checklist of items that should be included in reports of qualitative research. You must report the page number in your manuscript where you consider each of the items listed in this checklist. If you have not included this information, either revise your manuscript accordingly before submitting or note N/A.

Topic	Item No.	Guide Questions/Description	Reported on Page No.
**Domain 1: Research team and reflexivity**			
Personal characteristics			
Interviewer/facilitator	1	Which author/s conducted the interview or focus group?	
Credentials	2	What were the researcher's credentials? E.g. PhD, MD	
Occupation	3	What was their occupation at the time of the study?	
Gender	4	Was the researcher male or female?	
Experience and training	5	What experience or training did the researcher have?	
Relationship with participants			
Relationship established	6	Was a relationship established prior to study commencement?	
Participant knowledge of the interviewer	7	What did the participants know about the researcher? e.g. personal goals, reasons for doing the research	
Interviewer characteristics	8	What characteristics were reported about the inter viewer/facilitator? e.g. Bias, assumptions, reasons and interests in the research topic	
**Domain 2: Study design**			
Theoretical framework			
Methodological orientation and Theory	9	What methodological orientation was stated to underpin the study? e.g. grounded theory, discourse analysis, ethnography, phenomenology, content analysis	
Participant selection			
Sampling	10	How were participants selected? e.g. purposive, convenience, consecutive, snowball	
Method of approach	11	How were participants approached? e.g. face-to-face, telephone, mail, email	
Sample size	12	How many participants were in the study?	
Non-participation	13	How many people refused to participate or dropped out? Reasons?	
Setting			
Setting of data collection	14	Where was the data collected? e.g. home, clinic, workplace	
Presence of non-participants	15	Was anyone else present besides the participants and researchers?	
Description of sample	16	What are the important characteristics of the sample? e.g. demographic data, date	
Data collection			
Interview guide	17	Were questions, prompts, guides provided by the authors? Was it pilot tested?	
Repeat interviews	18	Were repeat inter views carried out? If yes, how many?	
Audio/visual recording	19	Did the research use audio or visual recording to collect the data?	
Field notes	20	Were field notes made during and/or after the inter view or focus group?	
Duration	21	What was the duration of the inter views or focus group?	
Data saturation	22	Was data saturation discussed?	
Transcripts returned	23	Were transcripts returned to participants for comment and/or	

For peer review only

Topic	Item No.	Guide Questions/Description	Reported on Page No.
		correction?	
**Domain 3: analysis and findings**			
Data analysis			
Number of data coders	24	How many data coders coded the data?	
Description of the coding tree	25	Did authors provide a description of the coding tree?	
Derivation of themes	26	Were themes identified in advance or derived from the data?	
Software	27	What software, if applicable, was used to manage the data?	
Participant checking	28	Did participants provide feedback on the findings?	
Reporting			
Quotations presented	29	Were participant quotations presented to illustrate the themes/findings? Was each quotation identified? e.g. participant number	
Data and findings consistent	30	Was there consistency between the data presented and the findings?	
Clarity of major themes	31	Were major themes clearly presented in the findings?	
Clarity of minor themes	32	Is there a description of diverse cases or discussion of minor themes?	

Developed from: Tong A, Sainsbury P, Craig J. Consolidated criteria for reporting qualitative research (COREQ): a 32-item checklist for interviews and focus groups. *International Journal for Quality in Health Care*. 2007. Volume 19, Number 6: pp. 349 – 357

Once you have completed this checklist, please save a copy and upload it as part of your submission. DO NOT include this checklist as part of the main manuscript document. It must be uploaded as a separate file.

For peer review only
